# Primary Trophoblast Cultures: Characterization of HLA Profiles and Immune Cell Interactions

**DOI:** 10.3389/fimmu.2022.814019

**Published:** 2022-05-13

**Authors:** Michael Eikmans, Carin van der Keur, Jacqueline D. H. Anholts, Jos J. M. Drabbels, Els van Beelen, Susana M. Chuva de Sousa Lopes, Marie-Louise van der Hoorn

**Affiliations:** ^1^ Department of Immunology, Leiden University Medical Center, Leiden, Netherlands; ^2^ Department of Anatomy and Embryology, Leiden University Medical Center, Leiden, Netherlands; ^3^ Department of Gynecology and Obstetrics, Leiden University Medical Center, Leiden, Netherlands

**Keywords:** trophoblast, HLA-G, placenta, pregnancy, culturing, stem cell, differentiation, immune cell

## Abstract

**Introduction:**

Trophoblasts are essential in fetal-maternal interaction during pregnancy. The goal was to study HLA profiles of primary trophoblasts derived from placentas, and to investigate their usefulness in studying interaction with immune cells.

**Methods:**

After enzymatic digestion of first-trimester placental tissue from seven donors (6-9 weeks gestation) and trophoblast enrichment we cultured cytotrophoblasts (CTB) in stem cell medium. CTB were differentiated into EVT in a Matrigel-containing medium. A subset of CTB/EVT was profiled for microRNA levels. Expression of classical HLA molecules and of HLA-G was studied by flow cytometry, qPCR, and ELISA. Secondary trophoblast cell lines JAR and JEG-3 were studied as controls. Lymphocytes were investigated during co-culturing with EVT.

**Results:**

The trophoblasts could be easily maintained for several passages, upregulated classical trophoblast markers (GATA3, TFAP2C, chromosome-19 microRNAs), and upon differentiation to EVT they were selective in expressing HLA-C. EVT showed increasing expression of total HLA-G, an increasing proportion of HLA-G1 over G2- and G3 isoforms, and elevated excretion of soluble HLA-G. These features were distinct from those of the secondary trophoblast cell lines. TNF-α and IL-8 represented the most abundantly secreted cytokines by CTB, but their levels were minimal in EVT cultures. As proof of principle, we showed that EVT affect lymphocytes in three-day co-cultures (n=4) by decreasing activation marker HLA-DR.

**Conclusion:**

We verified the possibility culturing trophoblasts from first-term placentas, and their capability of differentiating to HLA-G expressing EVT. This culture model better represents the *in-vivo* situation than previously studied secondary trophoblast cell lines and enables mechanistic studies of fetal-maternal interactions.

## Introduction

Trophoblasts play a significant role during pregnancy in fetal-maternal interaction. These cells are essential in the placentation process, and while invading in the uterine wall and the decidua of the mother they need to be tolerated by maternal immune cells. To study trophoblast characteristics and their interactions with other cell types, it is useful to establish a culture system after isolation of the trophoblasts. Following enzymatic digestion of placental tissue, trophoblasts may be directly enriched and cultured thereafter, but such primary cultures are relatively short-term due to limited proliferation capacity of the cells. Immortalized trophoblast cell lines including JEG-3, JAR, and HTR-8/SVneo are frequently used for *in vitro* research, but because of differences with primary trophoblasts (1–5) it is questionable whether such secondary cell lines are reliably modelling trophoblasts that are encountered *in vivo* (6). Placental explants in culture represent a more natural homologue to a placenta *in vivo*, but these explants show stress, cell death, and impaired nucleic acid content under certain conditions (7–11), which may confound results obtained in consecutive co-cultures.

The availability of long-term trophoblast cultures would circumvent the issues outlined above. Therefore, organoid cultures of trophoblasts from placentas at six to nine weeks of gestation have been generated, which were differentiated into EVT (12, 13). However, the drawback may be that three-dimensional organoids constitute multiple cell types, including villous trophoblasts and syncytiotrophoblast, and that these cell types display an inside-out orientation in comparison to villous tissue from the placenta. A possibly more appropriate model to study trophoblast-immune cell interactions may be a two-dimensional culture of one trophoblast cell type. A protocol was described to maintain cultured cytotrophoblasts (CTB) cultures, generated from trophoblast stem cells from first trimester placentas (14). The cell lines have the capacity to give rise to different trophoblast lineages, they show transcriptomes similar to those of primary trophoblast cells from the placenta, and they grow for at least one year, remaining genetically stable during that course (14).

Our objective was to establish 2D cultures of primary trophoblasts derived from first-term placentas, to study their profile of classical HLA molecules and HLA-G, and to examine if they could be used to study the interaction with immune cells.

## Materials and Methods

### Tissue Collection and Processing

Placental tissue was collected from donors (n=7) who had undergone termination of pregnancy due to social reasons between six and nine weeks of gestation. All samples were obtained after informed consent, and the study was carried out in accordance with the guidelines issued by the Medical Ethics Committee of the LUMC (P08.087) and in accordance with the Declaration of Helsinki.

Villous tissue was separated from the chorionic plate, minced, and washed three times with PBS (pH 7.4, Thermo Fisher Scientific, #AM9624). The tissue pellet was digested in 20-30 mL of 1:1 Accutase/TrypLE for 30 min at 37°C and filtered through a 70 µm-filter. The flow-through was taken up in 10 mL of Dulbecco’s Modified Eagle Medium (DMEM) containing 10% fetal bovine serum (FBS). After centrifugation for 5 min at 1,400 rpm, the pellet was taken up in 5 mL of DMEM/10% FBS and kept at 4°C. The lysate on the filter was digested again, followed by filtering and washing steps. This second pellet was pooled with the first pellet and centrifuged for 5 min at 1,500 rpm. The total pellet was resuspended in 1-2 mL of DMEM/10% FBS. A Percoll gradient was composed, containing 15 mL of 70% Percoll (Percoll SIP (Sigma-Aldrich/Merck/GE Healthcare, #17-0891-01) in PBS) and 20 mL of 15% Percoll (diluted in RPMI). The cell suspension was layered on this Percoll gradient and centrifuged for 30 min at 2,000 rpm with no brake. The trophoblast-containing interphase layer was separated, taken up in DMEM/10% FBS, and centrifuged for 5 min at 1,500 rpm. The pellet was washed in PBS/2% FBS/1 nM EDTA, taken up in 1 mL of the same buffer, and the number of cells were counted.

### Trophoblast Enrichment

Further enrichment of trophoblasts was established by magnetic bead retraction. For this, the EasySep human PE positive selection kit II was used (Stem Cell Technologies, #17684). All incubations were performed at room temperature in the dark: 100 µL of FcR blocker and 20 µL of PE-labeled ITGA6 (CD49f) antibody were added to the cells for 15 min. Further steps included addition of 30 µL of Selection Cocktail and 30 µL of RapidSpheres, both followed by 15 min of incubation. The step of bead retraction was repeated three times by replacing the flow-through each time again on the column. After the final step, cells were washed in PBS/2% FBS/1 nM EDTA and resuspended in TS-basal medium. This medium has been described previously (14) and contains DMEM/F-12 with 0.05 mM 2-mercapto-ethanol, 0.2% FBS, 1% penicillin/streptomycin (p/s), 0.3% bovine serum albumin (BSA), 0.5% KnockOut Serum Replacement (KSR), and 1% Insulin-Transferrin-Selenium (ITS)-X. A summary of reagents and growth factors is displayed in **Supplementary Table 1**.

### Culturing of CTB and Differentiation to EVT

Cells were cultured on collagen IV-coated plates in TS medium, which represents TS-basal medium (see above) supplemented with 1.5 μg/mL of L-ascorbic acid, 50 ng/mL of epithelial growth factor (EGF), 2 μM CHIR-99021, 0.5 μM A 83-01, 1 μM SB431542, 0.8 mM Valproic acid, and 5 μM Y-27632 (14). For coating of the plates, 5 µg/mL of collagen IV in PBS was incubated for 90 min at 37°C. After washing with PBS, 0.5 x 10^6^ CTB were incubated per 2 mL of TS medium at 37°C. During culturing the medium was refreshed every 2-3 days. Cells were transferred to a new, coated dish when they had reached a confluence of around 80%.

For phenotypic characterization of the basal CTB cultures, the cells were kept in culture for three days and subjected to flow cytometry, qPCR analysis, and Luminex analysis (see below). To harvest the cells, TS medium was removed and cells were washed by PBS. After addition of 1 mL of TrypLE reagent (to dissociate cells), the plate was incubated 15 min at 37°C and 1 mL of TS medium was added.

To study EVT, CTB cultures were harvested and 0.75 x 10^5^ cells were transferred to a fresh, collagen IV-coated (1 µg/mL) dish and incubated in 2 mL of EVT medium. This medium contains DMEM/F-12 supplemented with 0.1 mM 2-mercapto-ethanol, 0.5% p/s, 0.3% BSA, 1% ITS-X, 7.5 μM A 83-01, 2.5 μM Y-27632, 100 ng/ml of NRG1, and 4% KSR (14). At the end of resuspension, Matrigel (final concentration of 2%) was added. After three days, the medium was replaced with the same content but minus NRG1 and with 0.5% Matrigel. At six days, at approximately 80% confluence, the cells were either harvested for phenotypic characterization or transferred to a new, coated dish and incubated further for several days in EVT medium minus NRG1 and KSR and with 0.5% Matrigel. After this, cells and medium were subjected to characterization.

### Culturing of Immortalized Trophoblast Cell Lines and Mesenchymal Stromal Cells

As a control to the primary trophoblast cultures, the trophoblast cell lines JEG-3 and JAR were studied. These were cultured in IMDM with 10% FBS and p/s. In addition, fetal mesenchymal stromal cells (MSC) were studied, which were cultured on collagen-IV-coated plates in DMEM/F-12 supplemented with 10% FBS and p/s.

### Karyotyping

Three cell lines (QH1, QG1, RC2) were checked for DNA stability at later passages (17-18) by karyotyping, along with JAR and JEG-3. For this, 25 µL (250 ng) colcemid per mL of medium was added and incubated for 1 hour. Cells were then removed from the plate by trypsin/EDTA, fixed with methanol/acetic acid (3:1), incubated overnight at 60°C, and treated with Giemsa/Leishman stain to examine GTG banding on metaphase chromosomes from five cells per sample. Metaphases were analyzed on a GSL-120 platform (Leica) using CytoVision software.

### Genotyping

Typing of fetal tissues and maternal blood for the classical HLA molecules was performed at the National Reference Laboratory for Histocompatibility Testing at the LUMC, The Netherlands. Assessment of the fully phased *HLA-G* sequence was performed as described in a previous paper (15).

### Quantitative PCR for mRNA Analysis

RNA was extracted according to a previously described protocol (16). Quantitative PCR analysis was performed on a ViA7 in 15 µL-reactions containing 3 µL of 1:25 diluted cDNA, 0.3 µM primers, and 7.5 µL SybrGreen mix (Bio-Rad). Primer sequences are summarized in **Table 1**. The PCR program for *HLA-A*, *-B*, *-C*, and *DRB1* started with 95°C for 10 min, followed by 40 cycles of 95°C for 15 seconds, 68°C (decreasing one degree every cycle to 64°C) for 45 seconds, and 72°C for 30 seconds. All other markers were tested by the following program: 95°C for 10 min, followed by 40 cycles of 95°C for 15 seconds and 60°C for 1 min. Each PCR run was ended with a melting curve whereby the temperature was gradually increased from 55°C to 95°C at a rate of 0.05°C per second. Signals for the transcripts of interest were corrected for the average signal of two reference genes (*GAPDH* and *ACTB*) using the ΔCq calculation. For HLA-G-isoform-specific PCR assays, plasmid constructs containing either the G1, G2 or G3 isoform were tested as controls. These constructs were custom-made through GeneArt Gene Synthesis (Thermofisher Scientific).

**Table 1:** Overview of primers used for quantitative PCR assays in the study.

Target	Forward sequence	Reverse sequence
ACTB	ACCACACCTTCTACAATGAG	TAGCACAGCCTGGATAGC
CGA	AACCCATTCTTCTCCCAGCC	GTGGACTCTGAGGTGACGTT
ELF3	GGCCGATGACTTGGTACTGA	CAGCTCCTCAAGGGCACAAT
ELF5	TAAATCGGAAGCCCTGGCAA	ACTAACCTTCGGTCAACCCG
EPCAM	GAATGGCAAAGTATGAGAAGGCTGA	TCCCACGCACACACATTTGTAA
GAPDH	ACCCACTCCTCCACCTTTGAC	TCCACCACCCTGTTGCTGTAG
GATA3	CTCATTAAGCCCAAGCGAAG	GCATTCCTCCTCCAGAGTGT
HLA-A generic	TGGAGGAGGAAYAGCTCAGATA	CAAGGCAGCTGTCTCACAC
HLA-B generic	CCTAGCAGTTGTGGTCATC	AGCCCTGGGCACGGTCG
HLA-C generic	TGTCCTGGYTGTCCTAGCT	TGTCTCAGGCTTTACAAGYGA
HLA-C*01	CACAGACTGACCGAGTGAG	CCCCAGGTCGCAGCCAC
HLA-C*02/12	CCGCGGGTATGACCAGTC	CTCCAGGTAGGCTCTCCA
HLA-C*02/17	CGAGTGAACCTGCGGAAA	GAGCCACTCCACGCACTC
HLA-C*03	CACAGACTGACCGAGTGAG	AGCGTCTCCTTCCCATTCTT
HLA-C*04/18	CGAGTGAACCTGCGGAAA	GCCCCAGGTCGCAGCCAA
HLA-C*05	CGAGTGAACCTGCGGAAA	CGCGCGCTGCAGCGTCTT
HLA-C*06	TACTACAACCAGAGCGAGGA	GGTCGCAGCCATACATCCA
HLA-C*07	TACTACAACCAGAGCGAGGA	ACGGGCCGCCTCCA
HLA-C*07:03	TACTACAACCAGAGCGAGGA	GAGCCACTCCACGCACAG
HLA-C*08	ACGACACGCAGTTCGTGCA	GCGCAGGTTCCGCAGGC
HLA-C*14	CCACTCCATGAGGTATTTCTC	GGTCGCAGCCAAACATCCA
HLA-C*16	CCGCGGGTATGACCAGTC	CCCTCCAGGTAGGCTCTCT
HLA-C*18	AGTCCGAGAGGGGAGCCC	GCCCCAGGTCGCAGCCAA
HLA-DRB1 generic	ACAGTGGAATGGAGAGCACGG	CCAGAGTGTCCTTTCTGATTCCT
HLA-G generic	GACAGCGACTCGGCGT	GTGTTCCGTGTCTCCTCT
HLA-G1	ATGCTGCAGCGCGCGGACC	TGGGCAGGGAAGACTGCTTCCA
HLA-G2	ACCAGAGCGAGGCCAACCC	GGCAGGGAAGACTGCTTCCA
HLA-G3	AACCAGAGCGAGGCCAAGCAG	AGCTCCCTCCTTTTCAATCTGAG
ITGA1	TCAATGACTTTCAGCGGCCC	ACCTCTCCCAACTGGACACT
ITGA5	GGCTTCAACTTAGACGCGGA	GGCCGGTAAAACTCCACTGA
ITGA6	GAGCTTTTGTGATGGGCGATT	CTCTCCACCAACTTCATAAGGC
TEAD4	TGGACAAGCCCATCGACAAT	TGCCTGGTCCTTTAGCTTGG
TFAP2C	TGGACGAGGTGCAGAATGTC	TCAGTGGGGTTCATTACGGC

### MicroRNA Profiling

Thirteen samples (CTB/EVT from QG1, QH1, RF1; JAR; JEG-3; MSC from fetus and mother) were screened for approximately 750 microRNAs by real-time PCR using human miRNOME panels I+II (Exiqon, Vedbaek, Denmark). For each sample, 40 ng of RNA was reverse transcribed into cDNA using the Universal cDNA synthesis kit II (Exiqon) and dispersed over the wells. To correct for inter-assay variation, the samples were standardized to each other using the interplate controls (UniSp3) present on each plate. Next, input variations between samples were corrected using the geometric mean of four reference small-RNAs (423-3p, 423-5p, 103, U6), as indicated in the Exiqon guidelines. Then, detectable microRNAs were selected by including only the ones showing Cq < 37 in more than half of all samples.

### Flow Cytometry

To detect surface markers, cells were incubated with antibodies for 30 min on ice, washed with PBS/1% FBS, and incubated for 60 min in 1 mL of Fixation/Permeabilization diluent (ThermoFisher Scientific, 00-5123-43 and 00-5223) at room temperature. For intracellular stainings (VIM, KRT) cells were subsequently incubated in 2 mL of Permeabilization Buffer (ThermoFisher Scientific, 00-8333), followed by addition of the antibody, washing, and resuspension in PBS/1% FCS. An overview of antibodies for flow cytometry is summarized in **Table 2**.

**Table 2:** Overview of antibodies used for flow cytometry in the study^1^.

Protein	Fluorochrome	Company	Clone	Catalogue number
CD14	FITC	BD Bioscience	MФP9	345784
CD31	FITC	BioLegend	WM59	303104
CD45	APC	BioLegend	HI30	304012
CD56	Pacific Blue	BioLegend	HCD56	318325
CD73	BV421	Biolegend	AD2	344008
CD90/THY1	PE	Miltenyi Biotec	REA897	130-114-902
CDH1/CD324	Alexa-488	BioLegend	67A4	324110
EGFR/ERBB1	Brilliant Violet 421	BioLegend	AY13	352911
ERBB2	FITC	BioLegend	24D2	324404
ERBB3	PE	BioLegend	1B4C3	324706
HLA-A, B	Biotin	in-house developed HuMoAbs ^2^		
HLA-A/B/C	Pacific Blue	BioLegend	W6/32	311417
HLA-C	PE	BD Pharmingen	DT-9	566372
HLA-G	Alexa-488/PE	BioLegend	87G	335917
HLA-G	PE	Abcam	MEMG9	ab24384
HLA-G	FITC	Abcam	G233	ab7904-100
IgG1	Brilliant Violet 422	BioLegend	MCOP-21	400157
IgG1	Alexa-488	eBioscience	P3.6.2.8.1	53-4724-81
IgG1	PE	eBioscience	P3.6.2.8.1	12-4717-42
IgG2a (mouse)	Alexa-488	BioLegend	MOCP-173	400238
IgG2a (mouse)	Alexa-647	Biolegend	MOCP-173	400239
IgG2a (Rt)	PE	Biolegend	RTK2758	400508
IgG2a (mouse)	FITC	ThermoFisher	eBM2a	11-4724-81
IgG2b (mouse)	APC	BioLegend	27-35	402206
IgG2b (mouse)	PE	Biolegend	27-35	402204
ITGA1/CD49a	APC	BioLegend	TS2/7	328314
ITGA2/CD49b	APC	BioLegend	P1E6-C5	359309
ITGA2/CD49b	PE	BioLegend	P1E6-C5	359307
ITGA4/CD49d	PE	BioLegend	9F10	304303
ITGA5/CD49e	APC	BioLegend	NKI-SAM-1	328011
ITGA6/CD49f	PE	BioLegend	GoH3	313612
ITGB1/CD29	APC	BioLegend	TS2/16	303007
KRT	FITC	Miltenyi Biotec	CK-6H5	130-118-964
NOTCH1	APC	BioLegend	MHN1-519	352107
VIM	Alexa-647	BioLegend	O91D3	677807

^1^CDH1, cadherin-1; EGFR, epithelial growth factor receptor; KRT, cytokeratins; VIM, vimentin.

^2^HuMoAbs used were GV5D1 (A1/A9), SN607D8 (A2/A28), SN230G6 (A2/B57/58), BRO11F6 (A3/A11/A24), VTM1F11 (B7/B27/B60), BVK1F9 (B8), DK7C11 (B12), and HDG8D9 (B35/51), as described previously (17).

### Assessment of Proteins in the Supernatant

Culture medium of three CTB/EVT cultures (QG1, QH1, RC2) and control cell lines was subjected to assessment of soluble (s)HLA-G levels using an ELISA kit (MyBioSource, San Diego CA, #MBS2516229). Positive control in this assay was the HLA-G standard that was included in the kit, while MSC that are expected to not secrete sHLA-G functioned as negative control. Cytokines using a 17-plex Luminex assay were measured in four cultures (QG1, QG2, QH1, RG3). The procedure was carried as described previously (16). The following proteins were assessed: G-CSF, GM-CSF, IFN-γ, IL-1β, IL-2, IL-4, IL-5, IL-6, IL-7, IL-8, IL-10, IL-12-p70, IL-13, IL-17, MCP-1 (CCL2), MIP-1β (CCL4), and TNF-α. In addition, active TGF-β1, TGF-β2, and TGF-β3 were analyzed by single-assay Luminex.

### Co-culture of EVT and Immune Cells

CTB from QH1 were differentiated for six days to EVT. On the sixth day, 300,000 peripheral blood lymphocytes (PBL) of four different donors, obtained during pregnancy, were added to 150,000 EVT. This co-culture was maintained for three days in a 1:1 mixture of RPMI/HS/p/s/glut and EVT medium (see above). HLA-C genotypes of the PBL were C*03/C*07, C*04/C*07, C*07/C*07, and C*04/C*06. As control, PBL were cultured in the absence of EVT in either RPMI/HS/p/s/glut medium or in the 1:1 mixture with EVT medium. EVT remained attached to the plate, whereas immune cells remained floating. Hence, at the end of the co-culture immune cells were removed from the culture medium, gated for CD3 together with CD4 or CD8, and analyzed by flow cytometry for expression of CD69 and HLA-DR. These represent early and late T cell activation markers, respectively (18).

### Statistics

Difference in markers on T cells in absence or presence of EVT was calculated with paired samples t-tests. P<0.05 was considered statistically significant.

## Results

### Establishment of CTB and EVT Cultures and Success Rate

**Figure 1:**
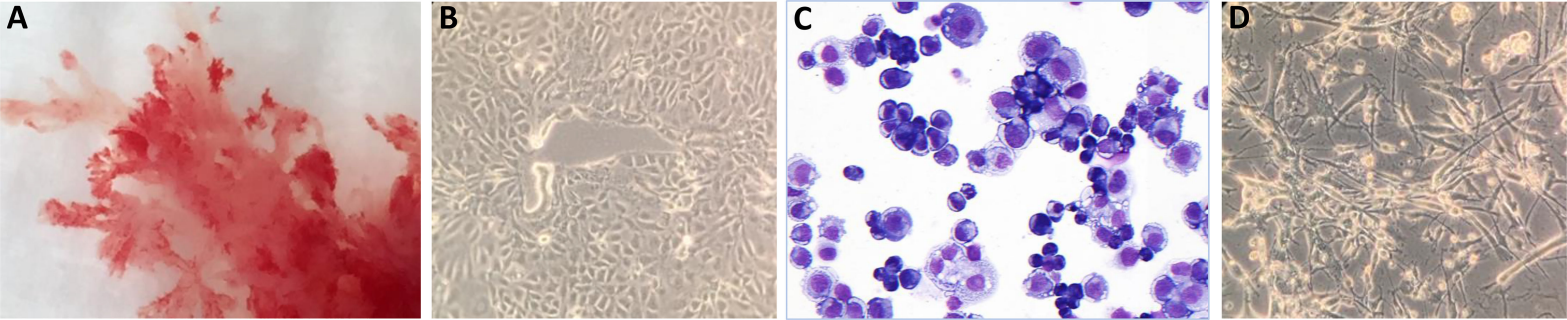
Macroscopic and microscopic pictures of trophoblast cells. **(A)** Cultured trophoblasts from a representative experiment (donor QH1) are shown. CTB were evaluated by **(B)** light microscopy or **(C)** after May Grunwald-Giemsa staining. The cells form a dense cluster, are rounded in structure and adhere to the plate. **(D)** Cells at the EVT stage have a more spindle-shaped appearance.

After digestion of the placental tissue and enrichment steps, trophoblasts were transferred to collagen-IV-coated culture plates in TS-basal medium. At a certain point, cultures were differentiated to EVT in specific medium containing Matrigel. Whereas cells at the CTB stage were forming a dense cluster, were rounded in structure and adhered to the plate, cells at the EVT stage had a more spindle-shaped appearance. Macroscopic and microscopic pictures of a representative successful experiment are shown in **Figure 1**.

Cells from a total of 12 donors were processed for culturing. Cultures from five donors were excluded from further study due to various reasons. Within the first weeks of culturing, cells from donor QI2 got infected and cells from donor PW1 were growing very slowly. Characterization of RC1 showed a considerable number of VIM-positive cells indicating contamination by mesenchymal stromal cells (MSC). Cells from PX1 still showed a circular morphology after six days of incubation in EVT differentiation medium. Cells from donor RF2 did not give interpretable results by genotyping. Hence, these five culture were excluded from further studies.

**Figure 2:**
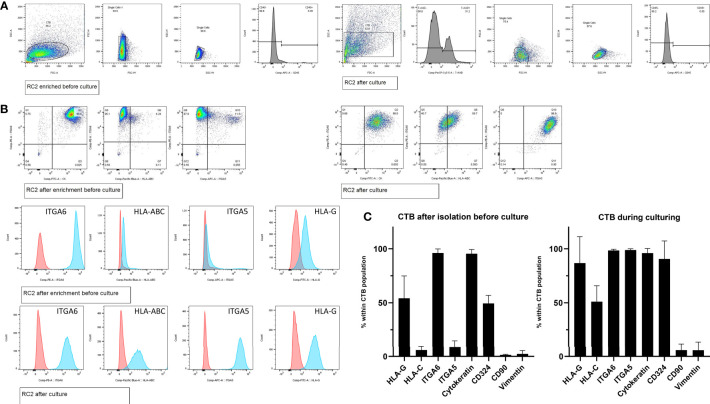
Cell frequencies by flow cytometry after enrichment and during CTB culturing. **(A)** Representative gating strategy from one donor (RC2), to select singlet cells by forward-sideward scatter that were viable (7-AAD^neg^) and non-hematopoietic (CD45^neg^). **(B)** Representative flow cytometry dot blots and histogram plots of trophoblasts from one donor (RC2), after cell enrichment but before culturing (upper panel) and during culturing in CTB stem cell medium (lower panel). **(C)** Mean cell frequencies from seven donors showing the positivity for each of the markers of interest, both after cell enrichment but before culturing (left graph) and during culturing in CTB stem cell medium (right graph).

Seven cultures were characterized for cell surface markers by flow cytometry. First, gating strategies were set by forward-sideward scatter, 7-AAD live-dead staining, and CD45 staining to select singlet, viable, and non-hematopoietic cells (**Figure 2A**). Whereas immediately after isolation most cells (>95%) were positive for ITGA6 and KRT with around 50% of the cells showing positivity of HLA-G and CDH1/CD324 and only the minority (<9%) being positive for ITGA5 and HLA-C, upon culturing more cells gained expression of HLA-C (51%), HLA-G (>88%), and CDH1/CD324 (>90%) (**Figures 2B, C**). The relatively high proportion of HLA-G^+^ cells after isolation probably may be explained because they mostly represented distal cell column trophoblasts, as detected in first-trimester placenta tissue (**Supplementary Figure 1A**). Secondly, HLA-G expression may have been driven by the high collagen IV content in the tissue surrounding the cells (**Supplementary Figure 1B**).

In general, we experienced the CTB to be easily maintained for many passages. Maintenance of chromosomal stability of these cells at later passages has been shown before (14), and here we confirmed this by karyotyping on three CTB lines at passage 17-18 (**Supplementary Figure 2**). QG1 was normal except for a slight addition on chromosome 17 in two out of five cells. We genotyped the cultures for the classical HLA molecules and for *HLA-G*. **Table 3** shows the overview of the *HLA-C* allele genotypes and the coding allele sequence and 3’-UTR haplotypes of *HLA-G*.

**Table 3:** Overview of the trophoblast cultures characterized in the study and their HLA-C and HLA-G genotypes^1, 2^.

Donor code	GA (weeks)	HLA-C genotype	HLA-G coding sequence	HLA-G 3’-UTR
QG1	6	C*07:01; C*07:04	G*01:01:02:01	UTR-2
QG2	9	C*07:01; C*07:02	G*01:01:01:01/G*01:01:01:05	UTR-1/UTR-4
QH1	9	C*07:01/02; C*07:02/10	G*01:01:01:01	UTR-1
RC2	6	C*01:02; C*05:01	G*01:01:01:05	UTR-4
RF1	6	C*03:04; C*16:01	G*01:01:01:01/G*01:04:01:01	UTR-1/UTR-3
RG2	6	C*04:01; C*07:01	G*01:01:03:03/G*01:03:01:02	UTR-7/UTR-17
RG3	8	C*02:10:02; C*03:03	G*01:01:01:01	UTR-1

^1^GA, gestational age; CDS, coding sequence; 3’-UTR, 3 prime untranslated region.

^2^Five out of 12 cultures were excluded from further study and not genotyped. QI2 and PW1 due to infection and poor growth, respectively, at the start of the culture; RC1 due to contamination by VIM-positive MSC; PX1 due to their circular morphology after EVT differentiation; and RF2 due to non-interpretable genotype results.

### Classical Trophoblast Markers

**Figure 3:**
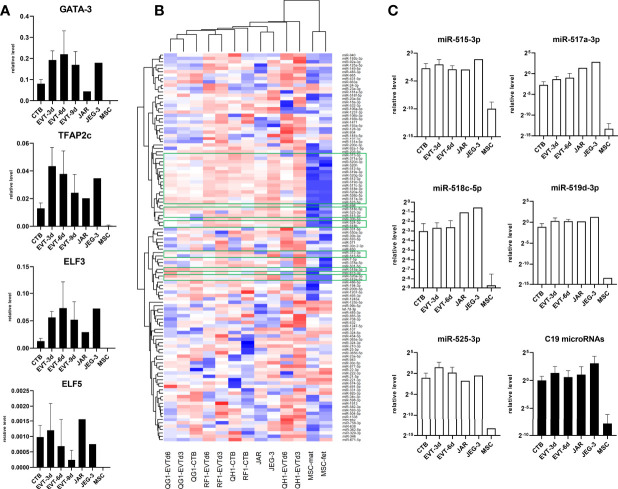
Expression of classical trophoblast markers in primary trophoblast cultures. **(A)** Messenger RNA expression of GATA3, TFAP2c, ELF3, and ELF5 (n = 6 primary cultures; n = 2 MSC cultures). **(B)** Approximately 750 microRNA were screened in primary CTB/EVT (n = 3), along with JAR, JEG-3, and two MSC lines. Out of these, 116 microRNAs were included for hierarchical clustering. Twenty-five C19 microRNA (green boxes) were typically expressed in trophoblasts. **(C)** Expression level of five typical C19 microRNAs (open bars) and average level of all C19 microRNAs per group (black bars). All graphs are represented by means with SD.

GATA3, TFAP2c, ELF3, and ELF5 have previously been identified as trophoblast markers (19, 20). Indeed, expression of these markers was high in primary CTB and EVT compared to negative controls (MSC), while expression in secondary trophoblast cell lines JAR and JEG-3 followed the same pattern (**Figure 3A**). We next screened for microRNAs in a subset of samples: 140 microRNAs could be detected, of which 116 showing the biggest differences between groups were included in hierarchical clustering (**Figure 3B**). Expression of microRNAs on chromosome (C)19 is a typical marker of trophoblasts (19, 21, 22), and indeed we found 25 C19 microRNAs to be elevated in primary CTB and EVT compared to controls (**Figure 3B**, green boxes; **Figure 3C**).

### CTB and EVT Express a Distinct Pattern of Cell Adhesion Markers

**Figure 4:**
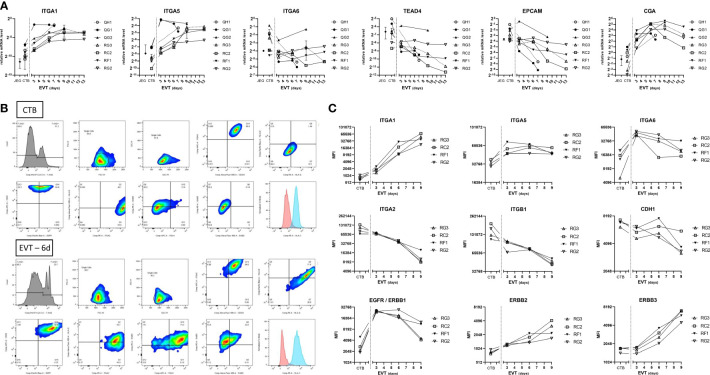
Expression of cell adhesion and epithelial markers in primary trophoblast cultures. **(A)** Expression of cell adhesion and trophoblast markers at the RNA level by culture days. Cultures (n = 7) were analyzed at the CTB and EVT phase for mRNA expression of *ITGA1*, *ITGA5*, *ITGA6*, *TEAD4*, *EPCAM*, and *CGA*. **(B)** Representative gating strategy from one donor (RC2), to select singlet cells by forward-sideward scatter that were viable (7-AAD^neg^). The figure shows dot blots of the markers studied in CTB (upper two panels) and EVT after 6 days of differentiation (lower two panels). **(C)** Expression of cell adhesion and epithelial markers at the protein level by culture days. Cultures (n = 4) were analyzed by flow cytometry at the CTB and EVT phase for surface expression of integrins, CDH1, and three members of the ErbB family.

As expression of integrins and epithelial/mesenchymal markers may help in clarifying the source, identity, and differentiation of trophoblasts (23–26), we next characterized the trophoblast cultures by qPCR analysis for different cell adhesion markers. Messenger RNA expression of integrins *ITGA1* and *ITGA5* increased when CTB were differentiated to EVT and reached a plateau from six days of differentiation onwards (**Figure 4A**). In contrast, *ITGA6* expression was relatively high in CTB and it decreased in EVT. TEAD4 and EPCAM have been described as CTB markers (12, 14, 27), and we found their mRNA expression indeed to be high in CTB cultures but decreased in EVT cultures. CGA is specific for syncytiotrophoblast (14, 27, 28), but we found its RNA expression also to be high in CTB, the level of which further increased in EVT (**Figure 4A**).

The results by qPCR for integrin markers were verified by flow cytometry on four cultures (**Figures 4B, C**), after establishing gating strategies by forward-sideward scatter and 7-AAD live-dead staining to select viable singlet cells (**Figure 4B**). ITGA1 and ITGA5 showed low and medium signals, respectively, in all CTB cultures and increased upon differentiation to EVT. ITGA6 signals were relatively high in all CTB cultures. Other integrins including ITGA2 and ITGB1 displayed homogeneous expression among the different trophoblast cultures, showing relatively high signals in CTB which progressively decreased during EVT differentiation. Similarly, cell adhesion molecule E-cadherin displayed relatively high expression on CTB, which decreased between three and nine days of EVT differentiation. Three members of the ErbB family, namely epidermal growth factor receptor (EGFR/ERBB1), ERBB2, and ERBB3, all showed relatively low signals by flow cytometry in CTB. EGFR signals peaked in day-3 EVT cultures and thereafter decreased. Both ERBB2 and ERBB3 showed increasing signals in EVT between three days and nine days of differentiation.

### Primary Trophoblasts Are Distinct From Immortalized Trophoblast Cell Lines and MSC in Cell Surface Markers

We further studied the trophoblast cell lines JEG-3 and JAR and compared them to primary trophoblast cultures with respect to cell surface markers. A control of fetal MSC was additionally included. The expression patterns of the different cell types have been summarized in **Table 4**. The two secondary cell lines were more similar in their expression pattern to primary CTB than to EVT. Only ITGA2 was dissimilar between the cell lines and the CTB. In contrast to primary trophoblasts, JEG-3 cells had low expression of HLA-C and HLA-G and JAR cells were devoid of these molecules. MSC typically expressed VIM and CD73, which were absent on trophoblasts. Furthermore, MSC moderately expressed ITGA1, ITGA6, and HLA class I.

**Table 4:** Surface markers on various cell cultures^1^.

Protein	CTB	EVT-d3	EVT-d6	EVT-d9	JEG-3	JAR	MSC
ITGA1	˗	++	++	+++	˗	˗	++
ITGA2	+++	++	+	+	±	˗	+++
ITGA5	++	+++	++	+++	+++	++	++
ITGA6	+++	++++	++	++	++++	++++	++
ITGB1	++++	+++	++	++	+++	++	++++
CDH1	+++	++	++	+	++	+++	±
KRT	+++	+++	++	+	++	++++	˗
EGFR/ERBB1	++	+++	++	+	+++	+++	++
ERBB2	±	+	++	+++	+	+	+
ERBB3	+	+	++	+++	+	+	˗
HLA-ABC	±	+	++	+++	+	˗	++
HLA-G	±	+	++	+++	±	˗	–
VIM	˗	˗	˗	˗	˗	˗	++++
CD73	˗	˗	˗	±	˗	˗	++++

^1^The intensity of antibody staining was scored – (negative), ± (minimal), + (low), ++ (medium), +++ (high) or ++++ (very high). MSC, mesenchymal stromal cells; CDH1, cadherin-1; KRT, cytokeratins; EGFR, epithelial growth factor receptor; VIM, vimentin.

### EVT Express HLA-C but No Other Classical HLA Molecules

**Figure 5:**
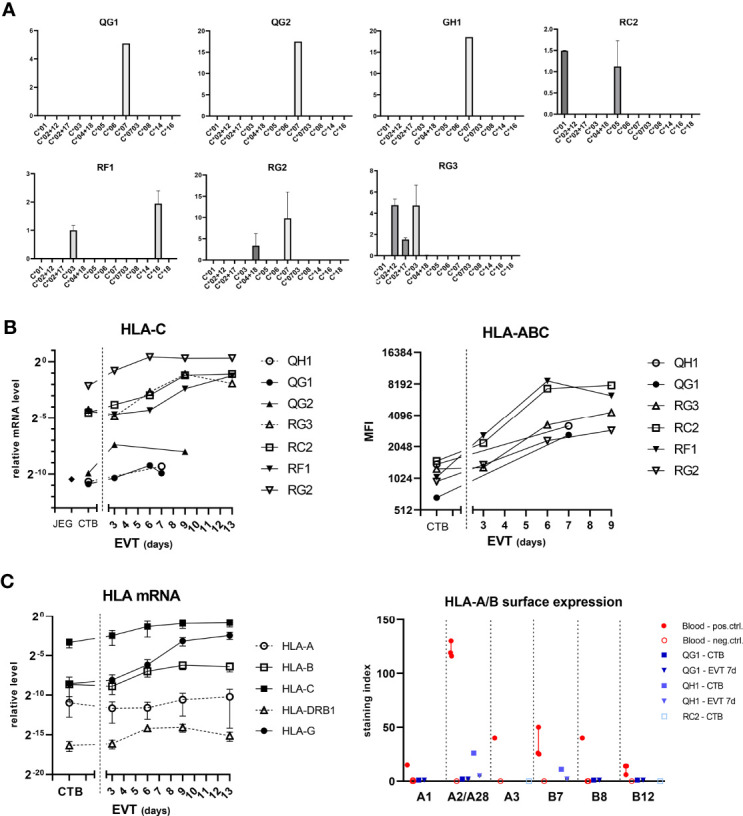
Expression of HLA-C in trophoblasts. **(A)** Messenger RNA expression of *HLA-C* according to the *HLA-C* alleles. RNA from CTB cultures (n = 7) was analyzed by quantitative PCR using primer sets that are specific for different *HLA-C* alleles. The vertical axes represent relative mRNA expression of *HLA-C* alleles, corrected for the average signal of two reference genes (*GAPDH*, *ACTB*). The graph shows means with SEM of two independent experiments per culture. **(B)** *HLA-C* mRNA expression was determined by quantitative PCR using generic primers that targeted all alleles (left panel). HLA-C was also studied by flow cytometry using antibodies against pan-HLA class I (right panel). **(C)** Messenger RNA expression of *HLA-A*, *-B*, *-C*, *-DRB1*, and *-G* (left panel) was studied in CTB and EVT cultures. RNA was analyzed by quantitative PCR using locus-specific primer sets. The graph shows means with SEM. Surface expression of individual HLA-A and HLA-B alleles was investigated by flow cytometry using human monoclonal antibodies on four blood cell samples and two trophoblast cultures (right panel). The graph displays medians with interquartile ranges.

We investigated expression of the classical HLA molecules in the trophoblast cultures (**Figure 5**). As the cells had been subjected to *HLA-C* genotyping (**Table 3**), we verified mRNA expression of *HLA-C* using allele-specific PCR primers. In each culture, positivity of mRNA expression was indeed only found for those alleles that had been found positive by DNA typing (**Figure 5A**). Upon differentiation to EVT, both *HLA-C* mRNA expression and surface expression of pan-HLA class I increased (**Figure 5B**). At the RNA level, out of all classical HLA loci *HLA-C* by far showed the most abundant expression both in CTB and EVT, the level being approximately eight, five, and 14 powers higher compared to that of *HLA-A*, *HLA-B*, and *HLA-DRB1*, respectively, on a log-2 scale (**Figure 5C**, left panel).

We next verified surface expression of individual HLA-A and -B alleles on two trophoblast cultures (QG1: HLA-A1/A68(28)/B8/B44(12); QH1: HLA-A2/A2/B7/B7) by flow cytometry using human monoclonal antibodies that were developed in-house, as previously described (29). Four different genotyped maternal blood samples were incorporated as controls to verify HLA antibody specificity (**Figure 5C**, right panel; **Supplementary Figure 3A**). JAR and JEG-3 cell lines were negative for HLA-A and -B (**Supplementary Figure 3B**). In EVT after seven days of differentiation, the surface index for HLA-A1, -A28, -B8, and -B12 in QG1 was around zero compared to blood samples (0-2 vs. 15, 119, 40, and 6-14, respectively) and expression of HLA-A2 and -B7 was minimal in QH1 (2-5 vs. 116-130 and 25-50, respectively; **Figure 5C**, right panel). We did observe in one CTB culture (QH1) some positivity for HLA-A2 (26.5) and -B7 (11.1), whereas the other CTB culture (QG1) was negative (A1: 0; A28: 1.7; B8: 0; B12: 0) (**Figure 5C** and **Supplementary Figure 3B**). Therefore, we tested CTB from a third donor, RC2 (HLA-A3/B44(12)/B56(22)). Both HLA-A3 and -B44 were negative by specific HLA antibodies in flow cytometry (**Supplementary Figure 3B**).

### EVT Upregulate HLA-G Expression and Secrete Soluble HLA-G

**Figure 6:**
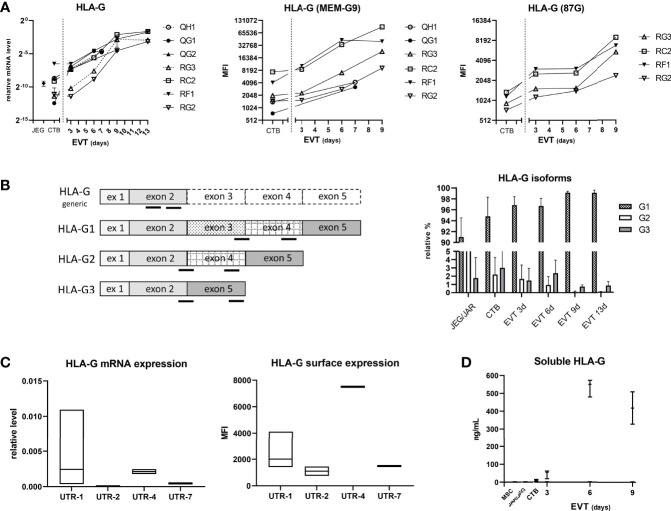
Expression of HLA-G in trophoblasts. **(A)** *HLA-G* mRNA expression was determined by quantitative PCR using generic primers that targeted all alleles (left panel). HLA-G was also studied by flow cytometry using antibodies against HLA class I and two epitopes on HLA-G1 and -G5 isoforms (MEM-G9 and 87G), respectively (middle and right panel). **(B)** *HLA-G1*, *G2*, and *G3* isoforms were separately studied by using isoform-specific primer sets (annealing locations indicated by black bars in the left panel). The results in the right panel are displayed as means with SD of n = 4 primary cultures. **(C)** HLA-G expression according to *HLA-G* 3’-UTR haplotype. Messenger RNA expression of *HLA-G* from seven CTB cultures was analyzed by quantitative PCR (corrected for the average signal of reference genes *GAPDH* and *ACTB*; left panel), whereas information of HLA-G surface expression by flow cytometry against MEMG9 was available for six CTB cultures (right panel). Expression data were grouped according to haplotype: UTR-1 (n = 4), UTR-2 (n = 2), UTR-4 (n = 2) or UTR-7 (n = 1). The graphs show floating bars (min to max with line at median). **(D)** Soluble HLA-G levels were determined by ELISA in supernatant of CTB (n = 3), EVT (n = 3), JEG-3 and JAR cell lines, and MSC (n = 2). The graph shows medians with the whiskers representing minimum and maximum values.

Next, we studied HLA-G expression in the trophoblasts (**Figure 6**). *HLA-G* mRNA expression progressively increased upon differentiation to EVT from three days to 13 days, and this observation was verified by flow cytometry using two different anti-HLA-G antibodies (MEM-G9 and 87G) (**Figure 6A**). For *HLA-G* mRNA analysis we developed primer sets that distinguished a single isoform, either *HLA-G1*, *G2* or *G3* (**Figure 6B**, left panel). Most of the *HLA-G* detected in CTB and the JEG-3 cell line consisted of the *G1* isoform; remarkably, with increasing time of differentiation to EVT the *G1* proportion further increased while the proportion of *G2* and *G3* decreased (**Figure 6B**, right panel). Furthermore, we analyzed relationship in the CTB cultures between the 3’-UTR haplotype of HLA-G and its expression and found that UTR-1 and UTR-4 haplotypes were related to relatively high expression and UTR-2 and UTR-7 with low expression (**Figure 6C**). Corresponding to the upregulated HLA-G expression by EVT, the supernatants of EVT, but not those from CTB, JEG-3/JAR cell lines, and MSC, displayed elevated levels of sHLA-G (20-40 ng/mL; **Figure 6D**).

### CTB and EVT Secrete Several Cytokines

**Figure 7:**
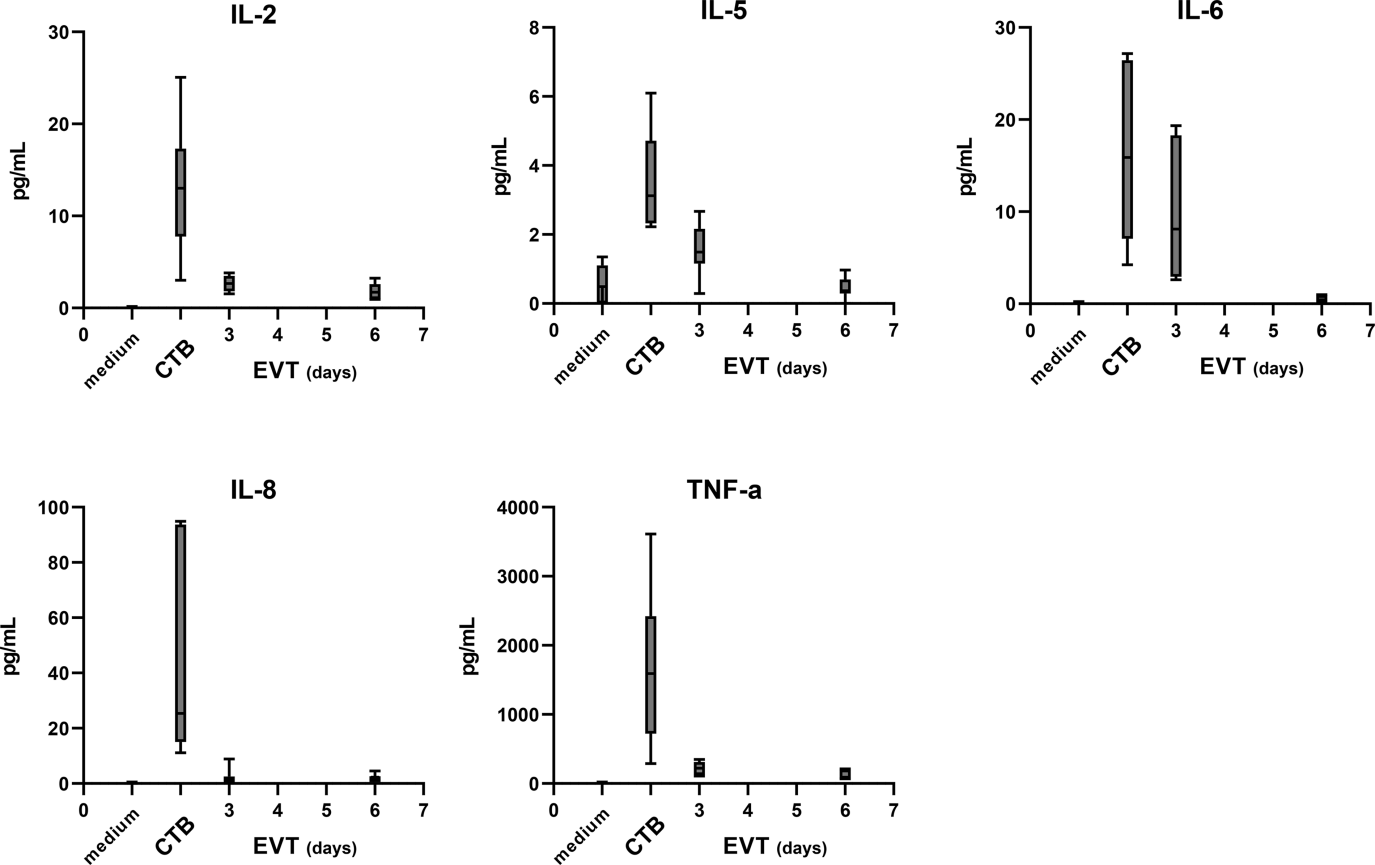
Cytokine secretion by trophoblasts in the culture medium. The level of 20 different cytokines and growth factors was analyzed by Luminex analysis in the medium from four cultures (QG1, QG2, RG3, and QH1 in triplicate). IL-2, IL-5, IL-6, IL-8, and TNF-α were detected above the minimum threshold value and are shown in this figure. The graph shows medians and the whiskers representing minimum and maximum values.

In addition to the sHLA-G levels, we analyzed 20 different cytokines and growth factors in the supernatants of CTB and EVT cultures, as these could also have a paracrine effect on immune cells upon interaction with trophoblasts. IL-2, IL-5, IL-6, IL-8, and TNF-α could be detected, of which the latter showed the highest levels. Generally, CTB showed the highest cytokine concentrations in the culture medium, and concentrations progressively decreased once the cells were differentiated to EVT (**Figure 7**).

### Co-Culturing of EVT and Immune Cells Leads to Decreased HLA-DR Expression on CD4^+^ T Cells

**Figure 8:**
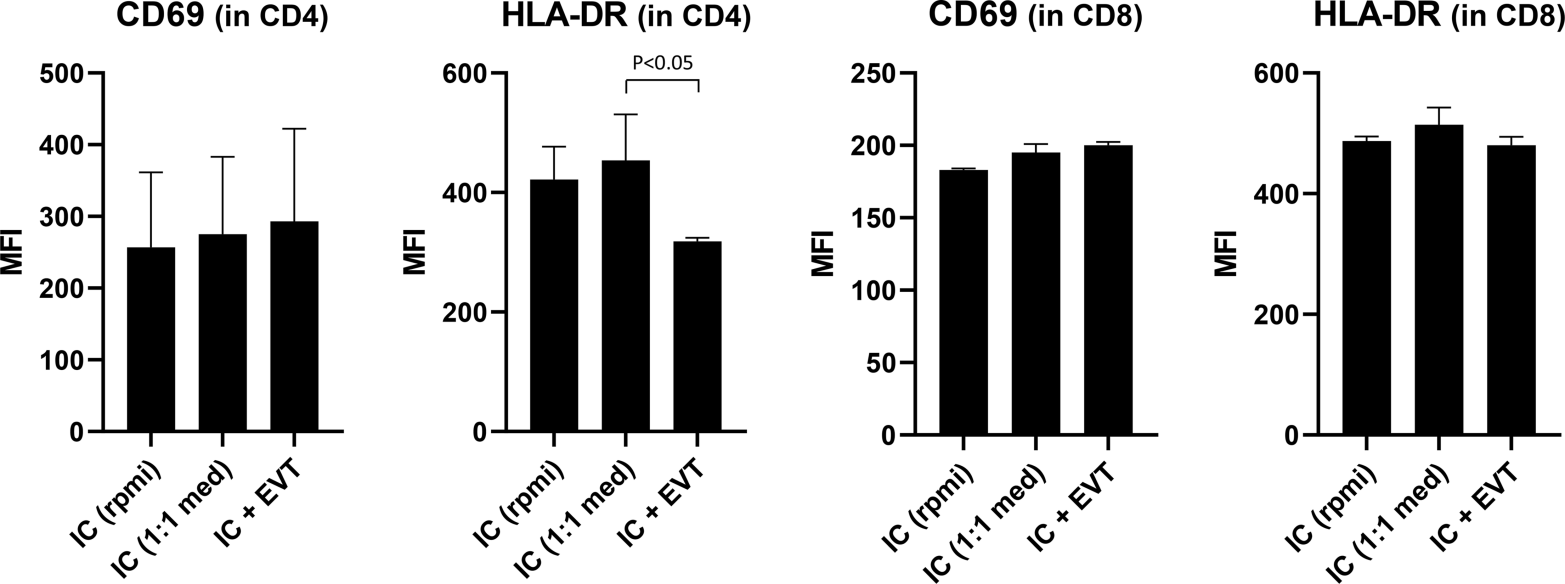
Expression of activation markers on CD4^+^ and CD8^+^ T cells following co-culturing of lymphocytes with EVT. First, EVT (culture QH1) were differentiated for six days. Then, lymphocytes from four different donors were added to the EVT for three days. HLA-DR and CD69 were studied by flow cytometry in both the CD3^+^CD4^+^ and CD3^+^CD8^+^ T cell population, both in the absence of EVT (first two columns) or in the presence of EVT (third column). The graph shows means with SEM. *P < 0.05.

Given our interest to use the trophoblast cultures as a model to study fetal-maternal interactions, as a proof of principle we investigated the effect of trophoblasts on immune cells. For this, we co-cultured four different sources of PBL together with EVT for three days. As control, PBL were cultured alone, either in their usual medium (RPMI plus supplements) or in a 1:1 mixture of EVT medium and RPMI medium. We evaluated two markers of T cell activation status, namely CD69 and HLA-DR. The composition of the medium did not affect expression of these markers on the T cells (**Figure 8**, first two bars of each graph). When cultured in the presence of EVT, CD4^+^ T cells showed a significant decrease (P<0.05) in HLA-DR expression on their surface (**Figure 8**, upper right graph). No differences were seen when lymphocytes were fully matched, mismatched at one allele or fully mismatched for HLA-C in comparison to the trophoblasts. The activation marker CD69 did not show a difference in CD4^+^ lymphocytes upon co-culturing. HLA-DR and CD69 expression on CD8^+^ T cells was not affected (**Figure 8**, lower panel).

## Discussion

For adequate study of the biology of trophoblasts and of their interaction with immune cells, stable trophoblast cultures are preferred. Here, we adopted a previously described protocol (14) for culturing CTB from first-term placental tissue and differentiating these to EVT. Isolation of trophoblasts from seven out of 12 donors (58.3%) led to successful results. Over the course of differentiation, trophoblasts upregulated HLA-C, HLA-G, ITGA1, and members of the ErbB family, and downregulated CDH1, ITGA2, and ITGB1. We verified that, like the *in-vivo* situation in the placenta, cultured EVT specifically express HLA-G and HLA-C but no other types of HLA molecules. In this way, the primary trophoblasts are clearly distinct from secondary, immortalized trophoblasts cell lines. Co-culturing of lymphocytes for three days with EVT led to decreased expression of HLA-DR on CD4^+^ T cells.

We did verify three out of four features typical of trophoblasts, as reviewed before (19, 30–32), in our cultures: expression of classical trophoblast genes (TFAP2C, GATA3), expression of chromosome 19 microRNAs, and specific upregulation of HLA-G and HLA-C during differentiation to EVT. ELF5 methylation was not tested, but despite of this we did find ELF5 expression to be specifically elevated in the trophoblasts. Expression of other markers that we studied in CTB and EVT corresponds with that of earlier findings. CTB typically express epithelial cell markers (CDH1, EPCAM, EGFR) (12, 14, 27, 33–35) and ITGA6 (33, 36), express the specific marker TEAD4 (14, 37), and also produce a considerable amount of CGA (37–41). Indeed, early studies have shown that not only syncytiotrophoblast but also EVT express and produce human chorionic gonadotropin, which promotes trophoblast invasion (42–44). The relatively high proportion of HLA-G^+^ cells directly after cell isolation from first-trimester placentas may be explained because they mostly represent distal cell column trophoblasts. We verified that these cells at the base of the cell column indeed were HLA-G positive by immunohistochemistry, and they have been shown to be ITGA6^+^ as well (45), similar to what we found in isolated cell fractions. The primary CTB cultures typically express ITGA2 and EPCAM, which suggests that the cells were derived from the cytotrophoblast column niche of the placenta, as put forward in previous studies (24, 46, 47). Of note, ITGA2^+^ cytotrophoblasts at the base of the cell column express medium levels of HLA-G and EGFR and relatively high levels of CDH1 (24), similar to what we found in CTB after isolation. Upon differentiation to EVT, the cells showed a spindle-shaped morphology, reminiscent of MSC and in line with the earlier described process of epithelial-to-mesenchymal transition (23). EVT show upregulation of HLA-G (14, 19, 27, 28, 38, 48–51), HLA-C (36, 52), ITGA1 (14, 25, 49, 51), and ITGA5 (14, 25, 33, 36, 49, 51, 53), whereas CDH1 is downregulated (33, 34). EVT express ERBB2 and ERBB3, which have been shown to be activated by NRG1 (54), a compound that is part of the EVT differentiation medium. In our study, a fair number of CTB during culture expressed ITGA5 and HLA-G, which showed further increase to only moderate extent at the EVT stage. These observations may be related to a certain degree of differentiation, as cells were cultured from the start on collagen-IV coated plates. Indeed, differentiation of CTB upon attachment to matrix molecules has been demonstrated before (36, 55, 56). With respect to HLA-A and HLA-B, interestingly a recent study showed detectable surface expression on CTB and EVT in the 2D trophoblast stem cell model by flow cytometry (47). Here, we did find moderate surface expression of these molecules on one of the CTB cultures studied. Nonetheless, we did not detect HLA-A and HLA-B expression whatsoever at the RNA and protein level in EVT, which represented the principal cell type that was used further in co-culture experiments.

Immortalized trophoblast cell lines including HTR-8/SVneo, JEG-3, and JAR are frequently used for *in vitro* research as a model of human placental physiology. The HTR-8/SVneo cell line was shown to represent not a homogenous collection of cells, but rather to contain two populations of cells, namely cytokeratin-7 positive trophoblasts and vimentin-positive mesenchymal cells (3). JEG-3 and JAR are human trophoblast cell lines derived from a choriocarcinoma tumor. Their transcriptomic profile is considerably divergent from that of primary trophoblasts (1, 2), and due to their tumorigenic nature the three cell lines display cytogenomic differences (4), suggesting that any results obtained from immortalized cell lines should be confirmed in primary trophoblasts. In the current study, JEG-3 and JAR were similar to primary CTB cultures for most of the cell adhesion markers studied, but in contrast to primary EVT cultures these cell lines expressed no or minimal amounts of HLA-C and HLA-G and did not secrete sHLA-G. Similar findings have been described before (5). Hence, these secondary cell lines are less suitable to be employed in co-culture studies.

Of the cytokines assessed in the supernatant of the trophoblast cultures, five could be detected above background levels, of which TNF-α and IL-8 showed the highest level for CTB. TNF-α positivity was found in EVT of placental bed biopsies at 10 weeks of gestation (57). IL-8 was shown to be produced by placental explants, and immunohistochemistry confirmed that trophoblasts are positive for IL-8 (58). One study determined 12 cytokines in culture medium of CTB and EVT derived from first-term placental tissue, whereby IL-8 showed the highest levels but TNF-α and IL-6 were not tested (59). Interestingly, while in their study cytokine levels were higher in EVT compared to villous CTB, we found the opposite. Another study profiled 92 proteins in conditioned medium from chorionic villous samples, and found IL-6 and IL-8 among the highest secreted but TNF-α one of the lowest ones (60).

In the current study, we presented results of a 2D trophoblast culture model. Previous studies have described establishment of a 3D organoid culture (12, 13, 47). The advantage of organoid cultures over 2D trophoblast stem cells may be that they represent more the organization of villous structures, as seen *in vivo*. The drawback is that they are more cumbersome to grow and expand, and that due to their higher structural complexity and inside-out orientation of CTB and SCT the effect following any interactions with immune cells is harder to dissect. This latter issue is expected to be more easily studyable in the 2D culture model. As described earlier (14), we experienced the trophoblast cultures to be easily frozen, stored, and thawed, in case they were needed for later culturing. Collection and processing of multiple placentas for this purpose generates a panel of trophoblast cell lines, which encompasses a diversity of HLA-C and HLA-G constitutions. Establishing a repository of trophoblast cultures with heterogeneous HLA surface molecules gives unique opportunity to study the effect of genetic variants in these molecules on expression and on outcome of cell-cell interactions. This became apparent for HLA-G where we found in the relatively small group of trophoblast cultures that UTR-1 and UTR-4 haplotypes are related to high expression and UTR-2 and UTR-7 are related to low expression. Similar haplotype-expression relationships have been previously found by us and others (61–64). Various allelic variants of HLA-G on EVT may interact differently with receptors on NK cells (65) and possibly with the ILT2 (LILRB1) receptor on T cells. Seven isoforms of HLA-G have been identified, which include four membrane-bound proteins (HLA-G1 to HLA-G4). Particularly HLA-G1 may be efficient in inhibiting proliferation and cytolysis of NK cells and cytotoxic T lymphocytes (66, 67). In this respect it is interesting that in the current study differentiating EVT showed increasing prevalence of the HLA-G1 isoform and decreasing percentage of HLA-G2 and -G3. HLA-G2 and G3 isoforms inhibit lysis by T cells to slightly lesser extent than the HLA-G1 isoform (68), whereas the effect on NK cell cytotoxicity is not consistent between studies (66, 68, 69). HLA-C represents the only classical class I molecule that is present on the EVT, and it may display a recognition and triggering signal for T cells. As a proof of principle, we tested the effect of the presence of EVT on blood lymphocytes by co-culturing for three days. This co-culture led to decreased expression of HLA-DR on the surface of CD4^+^ T cells. Here, we preferred cells from blood over those from first-trimester placentas, since the latter often represent small pieces of tissues containing relatively few lymphocytes and the T cells may already have been directed to a more memory/activation phenotype. Usage of blood PBL also more easily enabled to pick specific HLA genotypes in relation to the primary trophoblasts. Next studies would need to incorporate lymphocytes from the decidua to investigate cell-cell interactions. Further research is also needed to find out if the effect on T cells by trophoblasts is mediated by cell-cell contact or by the action of one or more soluble components, and by which mechanism HLA-DR expression is affected. Nevertheless, since a homogeneous response was observed among different lymphocyte cultures, irrespective of HLA genotype, we think the effect on T cells may have been caused by soluble factors secreted by EVT in the medium. In fact, a similar observation of decreased HLA-DR on CD4^+^ T cells was made by Svensson-Arvelund and colleagues after incubating blood CD4^+^ T cells for several days with conditioned medium from first-term placental explants (70).

One remark concerning the study setup needs to be made. With trophoblast cultures established from placentas of electively ended pregnancies it is not possible to know if the placenta would have developed properly with advancing gestation. One way to compensate for this lack of knowledge and to know if results represent a physiological behaviour would be to have sufficiently high sample numbers when studying trophoblast actions.

In summary, we verified the possibility of establishing trophoblast cultures from first-term placentas and their capability of differentiating to EVT. These primary trophoblast cultures specifically upregulate HLA-C and HLA-G on their surface upon differentiation and are clearly dissimilar from immortalized trophoblast cell lines. The primary trophoblast cultures described represent a suitable model system to further perform mechanistic studies of their interaction with immune cells.

## Data Availability Statement

The raw data supporting the conclusions of this article will be made available by the authors, without undue reservation.

## Ethics Statement

The studies involving human participants were reviewed and approved by Medical Ethics Committee of the LUMC (P08.087). The patients/participants provided their written informed consent to participate in this study.

## Author Contributions

ME analyzed and interpreted results and wrote down the manuscript. CK set up the cultures, performed flow cytometric analyses, and executed co-culture experiments. JA executed RNA isolations, cDNA analysis, and qPCR experiments. JD was involved in planning the genotyping experiments and interpreting their outcome. EB performed the Luminex assays. SC participated in coordinating the material transfer necessary for this study and in reviewing the content of the manuscript. M-LH participated in reviewing the clinical aspects and the content of the manuscript. All authors have read and approved the final version.

## Conflict of Interest

The authors declare that the research was conducted in the absence of any commercial or financial relationships that could be construed as a potential conflict of interest.

## Publisher’s Note

All claims expressed in this article are solely those of the authors and do not necessarily represent those of their affiliated organizations, or those of the publisher, the editors and the reviewers. Any product that may be evaluated in this article, or claim that may be made by its manufacturer, is not guaranteed or endorsed by the publisher.

## Supplementary Material

Supplementary Figure 1 Identification of distal cell column trophoblasts in placenta from electively ended pregnancy. **(A)** Tissue was obtained at first trimester and stained by hematoxylin-eosin (left picture) and for HLA-G (right picture). Proximal- and distal cell column trophoblast have been indicated in the figure. **(B)** First-trimester placental tissue was stained for HLA-G (left picture) and collagen IV (right picture).

Supplementary Figure 2 Karyotyping of trophoblast cell lines. Three primary lines (QG1, QH1, RC2) were analyzed at passage 16-17, along with secondary trophoblast lines JAR and JEG-3 as positive controls. No deviations were observed for QH1 (46,XX[5]) and RC2 (46,XY[5]). QG1 was also normal except for a small addition to chromosome 17 (inlay at lower right corner) in two out of five cells (46,XX[3]/46,XX,add(17)(q25)[2]). As comparison, JAR and JEG-3 (not shown) are broadly deviating.

Supplementary Figure 3 FACS histogram plots of human monoclonal HLA antibody stainings. **(A)** Four maternal blood samples were included as controls. **(B)** Expression of classical HLA class I antigens and HLA-G in three primary EVT cultures, in secondary trophoblast cell lines JEG-3 and JAR, and in maternal decidual stromal cells.

## Abbreviations

ACTB, Beta-actin; BSA, Bovine Serum Albumin; CDH1, Cadherin-1/E-cadherin; CGA, Chorionic Gonadotropin Subunit Alpha; CTB, Cytotrophoblasts; DMEM, Dulbecco’s Modified Eagle Medium; EDTA, Ethylenediaminetetraacetic Acid; EGFR, Epidermal Growth Factor Receptor; ELISA, Enzyme-linked Immunosorbent Assay; EPCAM, Epithelial Cell Adhesion Molecule; EVT, Extravillous Trophoblasts; FBS, Fetal Bovine Serum; GAPDH, Glyceraldehyde-3-Phosphate Dehydrogenase; HLA, Human Leukocyte Antigen; ITGA, Integrin Subunit Alpha; ITGB, Integrin Subunit Beta; IL, Interleukin; ITS, Insulin-Transferrin-Selenium; KSR, KnockOut Serum Replacement; KRT, Cytokeratins; MSC, Mesenchymal Stromal Cells; NRG1, Neuregulin-1; PBL, Peripheral Blood Lymphocytes; PBS, Phosphate-Buffered Saline; p/s, Penicillin/Streptomycin; SD, Standard Deviation; SEM, Standard Error of the Mean; TEAD4, TEA Domain Transcription Factor 4; TGF-β, Transforming Growth Factor, Beta; TNF-α, Tumor Necrosis Factor, Alpha; TS, Trophoblast Stem Cell; VIM, Vimentin.

## References

[1] Apps R Sharkey A Gardner L Male V Trotter M Miller N. Genome-Wide Expression Profile of First Trimester Villous and Extravillous Human Trophoblast Cells. Placenta (2011) 32:33–43. doi: 10.1016/j.placenta.2010.10.010 21075446 PMC3065343

[2] King A Thomas L Bischof P. Cell Culture Models of Trophoblast II: Trophoblast Cell Lines–a Workshop Report. Placenta (2000) 21 (Suppl A):S113–9. doi: 10.1053/plac.1999.0526 10831135

[3] Abou-Kheir W Barrak J Hadadeh O Daoud G. HTR-8/SVneo Cell Line Contains a Mixed Population of Cells. Placenta (2017) 50:1–7. doi: 10.1016/j.placenta.2016.12.007 28161053

[4] Weber M Weise A Vasheghani F Gohner C Fitzgerald JS Liehr T. Cytogenomics of Six Human Trophoblastic Cell Lines. Placenta (2021) 103:72–5. doi: 10.1016/j.placenta.2020.10.011 33096371

[5] Apps R Murphy SP Fernando R Gardner L Ahad T Moffett A. Human Leucocyte Antigen (HLA) Expression of Primary Trophoblast Cells and Placental Cell Lines, Determined Using Single Antigen Beads to Characterize Allotype Specificities of Anti-HLA Antibodies. Immunology (2009) 127:26–39. doi: 10.1111/j.1365-2567.2008.03019.x 19368562 PMC2678179

[6] Abbas Y Turco MY Burton GJ Moffett A. Investigation of Human Trophoblast Invasion In Vitro. Hum Reprod Update (2020) 26:501–13. doi: 10.1093/humupd/dmaa017 PMC7473396 32441309

[7] Di Santo S Malek A Sager R Andres AC Schneider H. Trophoblast Viability in Perfused Term Placental Tissue and Explant Cultures Limited to 7-24 Hours. Placenta (2003) 24:882–94. doi: 10.1016/S0143-4004(03)00142-5 13129686

[8] James JL Stone PR Chamley LW. The Effects of Oxygen Concentration and Gestational Age on Extravillous Trophoblast Outgrowth in a Human First Trimester Villous Explant Model. Hum Reprod (2006) 21:2699–705. doi: 10.1093/humrep/del212 16807282

[9] Reti NG Lappas M Huppertz B Riley C Wlodek ME Henschke P. Effect of High Oxygen on Placental Function in Short-Term Explant Cultures. Cell Tissue Res (2007) 328:607–16. doi: 10.1007/s00441-006-0375-1 17318588

[10] Brew O Nikolopoulou E Hughes A Christian M Lee Y Oduwole O. Quality of Placental RNA: Effects of Explant Size and Culture Duration. Placenta (2016) 46:45–8. doi: 10.1016/j.placenta.2016.08.083 27697221

[11] Brew O Sullivan MHF. Oxygen and Tissue Culture Affect Placental Gene Expression. Placenta (2017) 55:13–20. doi: 10.1016/j.placenta.2017.04.024 28623968

[12] Turco MY Gardner L Kay RG Hamilton RS Prater M Hollinshead MS. Trophoblast Organoids as a Model for Maternal-Fetal Interactions During Human Placentation. Nature (2018) 564:263–7. doi: 10.1038/s41586-018-0753-3 PMC7220805 30487605

[13] Haider S Meinhardt G Saleh L Kunihs V Gamperl M Kaindl U. Self-Renewing Trophoblast Organoids Recapitulate the Developmental Program of the Early Human Placenta. Stem Cell Rep (2018) 11:537–51. doi: 10.1016/j.stemcr.2018.07.004 PMC6092984 30078556

[14] Okae H Toh H Sato T Hiura H Takahashi S Shirane K. Derivation of Human Trophoblast Stem Cells. Cell Stem Cell (2018) 22:50–63.e6. doi: 10.1016/j.stem.2017.11.004 29249463

[15] Drabbels JJM Welleweerd R van Rooy I Johnsen GM Staff AC Haasnoot GW. HLA-G Whole Gene Amplification Reveals Linkage Disequilibrium Between the HLA-G 3’UTR and Coding Sequence. HLA (2020) 96:179–85. doi: 10.1111/tan.13909 PMC7384165 32307888

[16] Craenmehr MHC van der Keur C Anholts JDH Kapsenberg JM van der Westerlaken LA van Kooten C. Effect of Seminal Plasma on Dendritic Cell Differentiation *In Vitro* Depends on the Serum Source in the Culture Medium. J Reprod Immunol (2020) 137:103076. doi: 10.1016/j.jri.2019.103076 31981817

[17] Drabbels JJ de .Keur VC Kemps BM Mulder A Scherjon SA Claas FH. HLA-Targeted Flow Cytometric Sorting of Blood Cells Allows Separation of Pure and Viable Microchimeric Cell Populations. Blood (2011) 118:e149–55. doi: 10.1182/blood-2011-06-362053 21931111

[18] Caruso A Licenziati S Corulli M Canaris AD De Francesco MA Fiorentini S. Flow Cytometric Analysis of Activation Markers on Stimulated T Cells and Their Correlation With Cell Proliferation. Cytometry (1997) 27:71–6. doi: 10.1002/(SICI)1097-0320(19970101)27:1<71::AID-CYTO9>3.0.CO;2-O 9000587

[19] Lee CQ Gardner L Turco M Zhao N Murray MJ Coleman N. What Is Trophoblast? A Combination of Criteria Define Human First-Trimester Trophoblast. Stem Cell Rep (2016) 6:257–72. doi: 10.1016/j.stemcr.2016.01.006 PMC4750161 26862703

[20] Li Q Meissner TB Wang F Du Z Ma S Kshirsagar S. ELF3 Activated by a Superenhancer and an Autoregulatory Feedback Loop is Required for High-Level HLA-C Expression on Extravillous Trophoblasts. Proc Natl Acad Sci USA (2021) 118:1–9. doi: 10.1073/pnas.2025512118 PMC7936349 33622787

[21] Morales-Prieto DM Chaiwangyen W Ospina-Prieto S Schneider U Herrmann J Gruhn B. MicroRNA Expression Profiles of Trophoblastic Cells. Placenta (2012) 33:725–34. doi: 10.1016/j.placenta.2012.05.009 22721760

[22] Mong EF Yang Y Akat KM Canfield J VanWye J Lockhart J. Chromosome 19 microRNA Cluster Enhances Cell Reprogramming by Inhibiting Epithelial-to-Mesenchymal Transition. Sci Rep (2020) 10:3029. doi: 10.1038/s41598-020-59812-8 32080251 PMC7033247

[23] Davies JE Pollheimer J Yong HE Kokkinos MI Kalionis B Knofler M. Epithelial-Mesenchymal Transition During Extravillous Trophoblast Differentiation. Cell Adh Migr (2016) 10:310–21. doi: 10.1080/19336918.2016.1170258 PMC4951171 27070187

[24] Lee CQE Turco MY Gardner L Simons BD Hemberger M Moffett A. Integrin Alpha2 Marks a Niche of Trophoblast Progenitor Cells in First Trimester Human Placenta. Development (2018) 145:1–9. doi: 10.1242/dev.162305 PMC6124543 29540503

[25] Damsky CH Librach C Lim KH Fitzgerald ML McMaster MT Janatpour M. Integrin Switching Regulates Normal Trophoblast Invasion. Development (1994) 120:3657–66. doi: 10.1242/dev.120.12.3657 7529679

[26] DaSilva-Arnold S James JL Al-Khan A Zamudio S Illsley NP. Differentiation of First Trimester Cytotrophoblast to Extravillous Trophoblast Involves an Epithelial-Mesenchymal Transition. Placenta (2015) 36:1412–8. doi: 10.1016/j.placenta.2015.10.013 26545962

[27] Liu Y Fan X Wang R Lu X Dang YL Wang H. Single-Cell RNA-Seq Reveals the Diversity of Trophoblast Subtypes and Patterns of Differentiation in the Human Placenta. Cell Res (2018) 28:819–32. doi: 10.1038/s41422-018-0066-y PMC6082907 30042384

[28] Tsang JCH Vong JSL Ji L Poon LCY Jiang P Lui KO. Integrative Single-Cell and Cell-Free Plasma RNA Transcriptomics Elucidates Placental Cellular Dynamics. Proc Natl Acad Sci USA (2017) 114:E7786–95. doi: 10.1073/pnas.1710470114 PMC5604038 28830992

[29] Mulder A Kardol M Regan J Buelow R Claas F. Reactivity of Twenty-Two Cytotoxic Human Monoclonal HLA Antibodies Towards Soluble HLA Class I in an Enzyme-Linked Immunosorbent Assay (PRA-STAT). Hum Immunol (1997) 56:106–13. doi: 10.1016/S0198-8859(97)00146-8 9455499

[30] Silva JF Serakides R. Intrauterine Trophoblast Migration: A Comparative View of Humans and Rodents. Cell Adh Migr (2016) 10:88–110. doi: 10.1080/19336918.2015.1120397 26743330 PMC4853047

[31] Knofler M Haider S Saleh L Pollheimer J Gamage T James J. Human Placenta and Trophoblast Development: Key Molecular Mechanisms and Model Systems. Cell Mol Life Sci (2019) 76:3479–96. doi: 10.1007/s00018-019-03104-6 PMC6697717 31049600

[32] Io S Kondoh E Chigusa Y Kawasaki K Mandai M Yamada AS. New Era of Trophoblast Research: Integrating Morphological and Molecular Approaches. Hum Reprod Update (2020) 26:611–33. doi: 10.1093/humupd/dmaa020 32728695

[33] Robinson JF Kapidzic M Gormley M Ona K Dent T Seifikar H. Transcriptional Dynamics of Cultured Human Villous Cytotrophoblasts. Endocrinology (2017) 158:1581–94. doi: 10.1210/en.2016-1635 PMC5460928 28323933

[34] Harris LK Jones CJ Aplin JD. Adhesion Molecules in Human Trophoblast - a Review. II. Extravillous Trophoblast. Placenta (2009) 30:299–304. doi: 10.1016/j.placenta.2008.12.003 19131105

[35] Aplin JD Jones CJ Harris LK. Adhesion Molecules in Human Trophoblast - a Review. I. Villous Trophoblast. Placenta (2009) 30:293–8. doi: 10.1016/j.placenta.2008.12.001 19131106

[36] Tilburgs T Crespo AC van der Zwan A Rybalov B Raj T Stranger B. Human HLA-G+ Extravillous Trophoblasts: Immune-Activating Cells That Interact With Decidual Leukocytes. Proc Natl Acad Sci USA (2015) 112:7219–24. doi: 10.1073/pnas.1507977112 PMC4466754 26015573

[37] Chen Y Wang K Chandramouli GV Knott JG Leach R. Trophoblast Lineage Cells Derived From Human Induced Pluripotent Stem Cells. Biochem Biophys Res Commun (2013) 436:677–84. doi: 10.1016/j.bbrc.2013.06.016 PMC3750115 23774580

[38] Pavlicev M Wagner GP Chavan AR Owens K Maziarz J Dunn-Fletcher C. Single-Cell Transcriptomics of the Human Placenta: Inferring the Cell Communication Network of the Maternal-Fetal Interface. Genome Res (2017) 27:349–61. doi: 10.1101/gr.207597.116 PMC5340963 28174237

[39] Genbacev O Donne M Kapidzic M Gormley M Lamb J Gilmore J. Establishment of Human Trophoblast Progenitor Cell Lines From the Chorion. Stem Cells (2011) 29:1427–36. doi: 10.1002/stem.686 PMC3345889 21755573

[40] Marchand M Horcajadas JA Esteban FJ McElroy SL Fisher SJ Giudice LC. Transcriptomic Signature of Trophoblast Differentiation in a Human Embryonic Stem Cell Model. Biol Reprod (2011) 84:1258–71. doi: 10.1095/biolreprod.110.086413 21368299

[41] Xu RH Chen X Li DS Li R Addicks GC Glennon C. BMP4 Initiates Human Embryonic Stem Cell Differentiation to Trophoblast. Nat Biotechnol (2002) 20:1261–4. doi: 10.1038/nbt761 12426580

[42] Handschuh K Guibourdenche J Cocquebert M Tsatsaris V Vidaud M Evain-Brion D. Expression and Regulation by PPARgamma of hCG Alpha- and Beta-Subunits: Comparison Between Villous and Invasive Extravillous Trophoblastic Cells. Placenta (2009) 30:1016–22. doi: 10.1016/j.placenta.2009.09.006 19846218

[43] Handschuh K Guibourdenche J Tsatsaris V Guesnon M Laurendeau I Evain-Brion D. Human Chorionic Gonadotropin Produced by the Invasive Trophoblast But Not the Villous Trophoblast Promotes Cell Invasion and is Down-Regulated by Peroxisome Proliferator-Activated Receptor-Gamma. Endocrinology (2007) 148:5011–9. doi: 10.1210/en.2007-0286 17628005

[44] Handschuh K Guibourdenche J Tsatsaris V Guesnon M Laurendeau I Evain-Brion D. Human Chorionic Gonadotropin Expression in Human Trophoblasts From Early Placenta: Comparative Study Between Villous and Extravillous Trophoblastic Cells. Placenta (2007) 28:175–84. doi: 10.1016/j.placenta.2006.01.019 16584772

[45] Tabrizi MEA Lancaster TL Ismail TM Georgiadou A Ganguly A Mistry JJ. S100P Enhances the Motility and Invasion of Human Trophoblast Cell Lines. Sci Rep (2018) 8:11488. doi: 10.1038/s41598-018-29852-2 30065265 PMC6068119

[46] Cinkornpumin JK Kwon SY Guo Y Hossain I Sirois J Russett CS. Naive Human Embryonic Stem Cells Can Give Rise to Cells With a Trophoblast-Like Transcriptome and Methylome. Stem Cell Rep (2020) 15:198–213. doi: 10.1016/j.stemcr.2020.06.003 PMC7363941 32619492

[47] Sheridan MA Zhao X Fernando RC Gardner L Perez-Garcia V Li Q. Characterization of Primary Models of Human Trophoblast. Development (2021) 148:1–13. doi: 10.1242/dev.199749 PMC8602945 34651188

[48] Suryawanshi H Morozov P Straus A Sahasrabudhe N Max KEA Garzia A. A Single-Cell Survey of the Human First-Trimester Placenta and Decidua. Sci Adv (2018) 4:eaau4788. doi: 10.1126/sciadv.aau4788 30402542 PMC6209386

[49] Meinhardt G Haider S Haslinger P Proestling K Fiala C Pollheimer J. Wnt-Dependent T-Cell Factor-4 Controls Human Etravillous Trophoblast Motility. Endocrinology (2014) 155:1908–20. doi: 10.1210/en.2013-2042 24605829

[50] James JL Stone PR Chamley LW. The Isolation and Characterization of a Population of Extravillous Trophoblast Progenitors From First Trimester Human Placenta. Hum Reprod (2007) 22:2111–9. doi: 10.1093/humrep/dem144 17580299

[51] Nagamatsu T Fujii T Ishikawa T Kanai T Hyodo H Yamashita T. A Primary Cell Culture System for Human Cytotrophoblasts of Proximal Cytotrophoblast Cell Columns Enabling *In Vitro* Acquisition of the Extra-Villous Phenotype. Placenta (2004) 25:153–65. doi: 10.1016/j.placenta.2003.08.015 14972448

[52] King A Burrows TD Hiby SE Bowen JM Joseph S Verma S. Surface Expression of HLA-C Antigen by Human Extravillous Trophoblast. Placenta (2000) 21:376–87. doi: 10.1053/plac.1999.0496 10833373

[53] Chang WL Liu YW Dang YL Jiang XX Xu H Huang X. PLAC8, a New Marker for Human Interstitial Extravillous Trophoblast Cells, Promotes Their Invasion and Migration. Development (2018) 145:1–11. doi: 10.1242/dev.148932 PMC5825838 29361555

[54] Fock V Plessl K Draxler P Otti GR Fiala C Knofler M. Neuregulin-1-Mediated ErbB2-ErbB3 Signalling Protects Human Trophoblasts Against Apoptosis to Preserve Differentiation. J Cell Sci (2015) 128:4306–16. doi: 10.1242/jcs.176933 PMC4712818 26490994

[55] Damsky CH Fitzgerald ML Fisher SJ. Distribution Patterns of Extracellular Matrix Components and Adhesion Receptors are Intricately Modulated During First Trimester Cytotrophoblast Differentiation Along the Invasive Pathway, In Vivo. J Clin Invest (1992) 89:210–22. doi: 10.1172/JCI115565 PMC442839 1370295

[56] Aplin JD Haigh T Jones CJ Church HJ Vicovac L. Development of Cytotrophoblast Columns From Explanted First-Trimester Human Placental Villi: Role of Fibronectin and Integrin Alpha5beta1. Biol Reprod (1999) 60:828–38. doi: 10.1095/biolreprod60.4.828 10084955

[57] Otun HA Lash GE Innes BA Bulmer JN Naruse K Hannon T. Effect of Tumour Necrosis Factor-Alpha in Combination With Interferon-Gamma on First Trimester Extravillous Trophoblast Invasion. J Reprod Immunol (2011) 88:1–11. doi: 10.1016/j.jri.2010.10.003 21112094

[58] Shimoya K Matsuzaki N Taniguchi T Kameda T Koyama M Neki R. Human Placenta Constitutively Produces Interleukin-8 During Pregnancy and Enhances Its Production in Intrauterine Infection. Biol Reprod (1992) 47:220–6. doi: 10.1095/biolreprod47.2.220 1391327

[59] Naruse K Innes BA Bulmer JN Robson SC Searle RF Lash GE. Secretion of Cytokines by Villous Cytotrophoblast and Extravillous Trophoblast in the First Trimester of Human Pregnancy. J Reprod Immunol (2010) 86:148–50. doi: 10.1016/j.jri.2010.04.004 20888997

[60] Bruno V Lindau R Jenmalm MC Ticconi C Piccione E Pietropolli A. First-Trimester Trophoblasts Obtained by Chorionic Villus Sampling Maintain Tolerogenic and Proteomic Features in Successful Pregnancies Despite a History of Unexplained Recurrent Pregnancy Loss. Am J Reprod Immunol (2020) 84:e13314. doi: 10.1111/aji.13314 32734710

[61] Craenmehr MHC Haasnoot GW Drabbels JJM Spruyt-Gerritse MJ Cao M van der Keur C. Soluble HLA-G Levels in Seminal Plasma are Associated With HLA-G 3’UTR Genotypes and Haplotypes. HLA (2019) 94:339–46. doi: 10.1111/tan.13628 PMC6772099 31321883

[62] Martelli-Palomino G Pancotto JA Muniz YC Mendes-Junior CT Castelli EC Massaro JD. Polymorphic Sites at the 3’ Untranslated Region of the HLA-G Gene are Associated With Differential Hla-G Soluble Levels in the Brazilian and French Population. PloS One (2013) 8:e71742. doi: 10.1371/journal.pone.0071742 24204558 PMC3808361

[63] Poras I Yaghi L Martelli-Palomino G Mendes-Junior CT Muniz YC Cagnin NF. Haplotypes of the HLA-G 3’ Untranslated Region Respond to Endogenous Factors of HLA-G+ and HLA-G- Cell Lines Differentially. PloS One (2017) 12:e0169032. doi: 10.1371/journal.pone.0169032 28045999 PMC5207740

[64] Dahl M Klitkou L Christiansen OB Djurisic S Piosik ZM Skovbo P. Human Leukocyte Antigen (HLA)-G During Pregnancy Part II: Associations Between Maternal and Fetal HLA-G Genotypes and Soluble HLA-G. Hum Immunol (2015) 76:260–71. doi: 10.1016/j.humimm.2015.01.015 25637667

[65] Celik AA Simper GS Huyton T Blasczyk R Bade-Doding C. HLA-G Mediated Immune Regulation Is Impaired by a Single Amino Acid Exchange in the Alpha 2 Domain. Hum Immunol (2018) 79:453–62. doi: 10.1016/j.humimm.2018.03.010 29605689

[66] Rouas-Freiss N Marchal RE Kirszenbaum M Dausset J Carosella ED. The Alpha1 Domain of HLA-G1 and HLA-G2 Inhibits Cytotoxicity Induced by Natural Killer Cells: Is HLA-G the Public Ligand for Natural Killer Cell Inhibitory Receptors? Proc Natl Acad Sci USA (1997) 94:5249–54. doi: 10.1073/pnas.94.10.5249 PMC24664 9144223

[67] Bainbridge DR Ellis SA Sargent IL. The Short Forms of HLA-G are Unlikely to Play a Role in Pregnancy Because They Are Not Expressed at the Cell Surface. J Reprod Immunol (2000) 47:1–16. doi: 10.1016/S0165-0378(00)00056-5 10779586

[68] Riteau B Rouas-Freiss N Menier C Paul P Dausset J Carosella ED. HLA-G2, -G3, and -G4 Isoforms Expressed as Nonmature Cell Surface Glycoproteins Inhibit NK and Antigen-Specific CTL Cytolysis. J Immunol (2001) 166:5018–26. doi: 10.4049/jimmunol.166.8.5018 11290782

[69] Zhao L Teklemariam T Hantash BM. Mutated HLA-G3 Localizes to the Cell Surface But Does Not Inhibit Cytotoxicity of Natural Killer Cells. Cell Immunol (2014) 287:23–6. doi: 10.1016/j.cellimm.2013.11.005 24355712

[70] Svensson-Arvelund J Mehta RB Lindau R Mirrasekhian E Rodriguez-Martinez H Berg G. The Human Fetal Placenta Promotes Tolerance Against the Semiallogeneic Fetus by Inducing Regulatory T Cells and Homeostatic M2 Macrophages. J Immunol (2015) 194:1534–44. doi: 10.4049/jimmunol.1401536 25560409

