# Supplementary material for: Primary Trophoblast Cultures: Characterization of HLA Profiles and Immune Cell Interactions
Source: Front Immunol. 2022 May 13;13:814019. doi: 10.3389/fimmu.2022.814019 (PMC9136060; doi:10.3389/fimmu.2022.814019)

Supplementary Figure 1

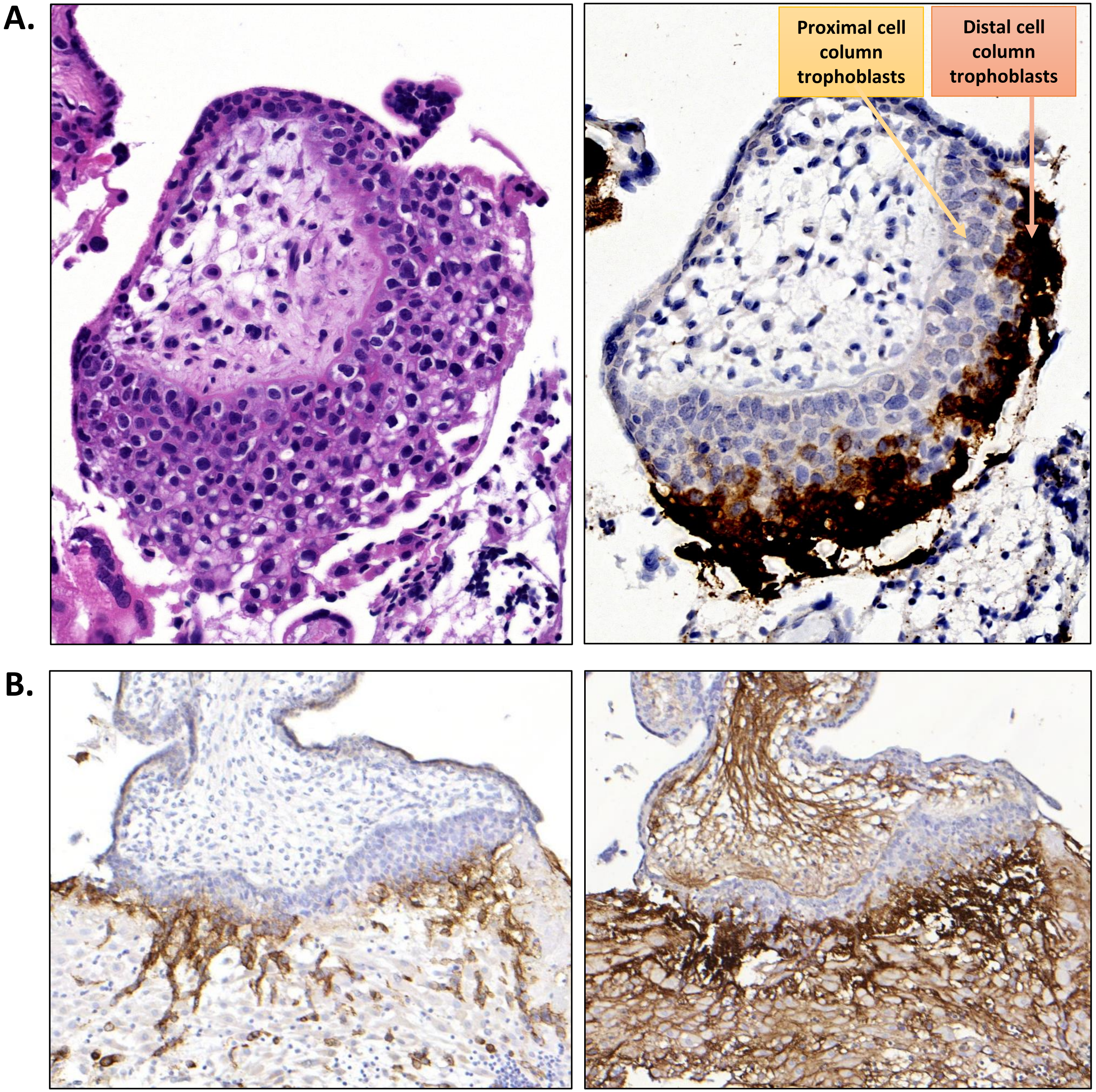

Supplementary Figure 2

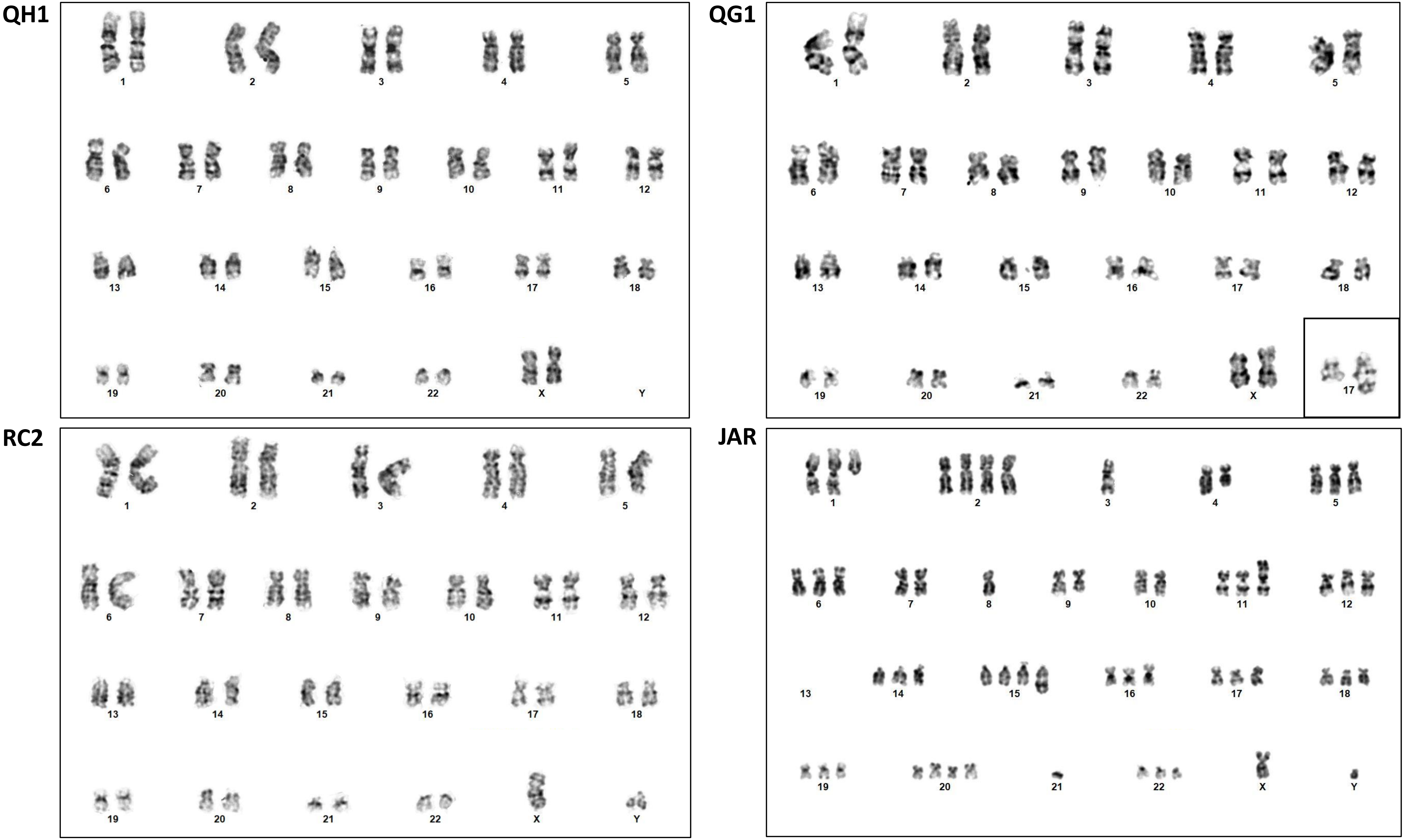

Supplementary Figure 3a

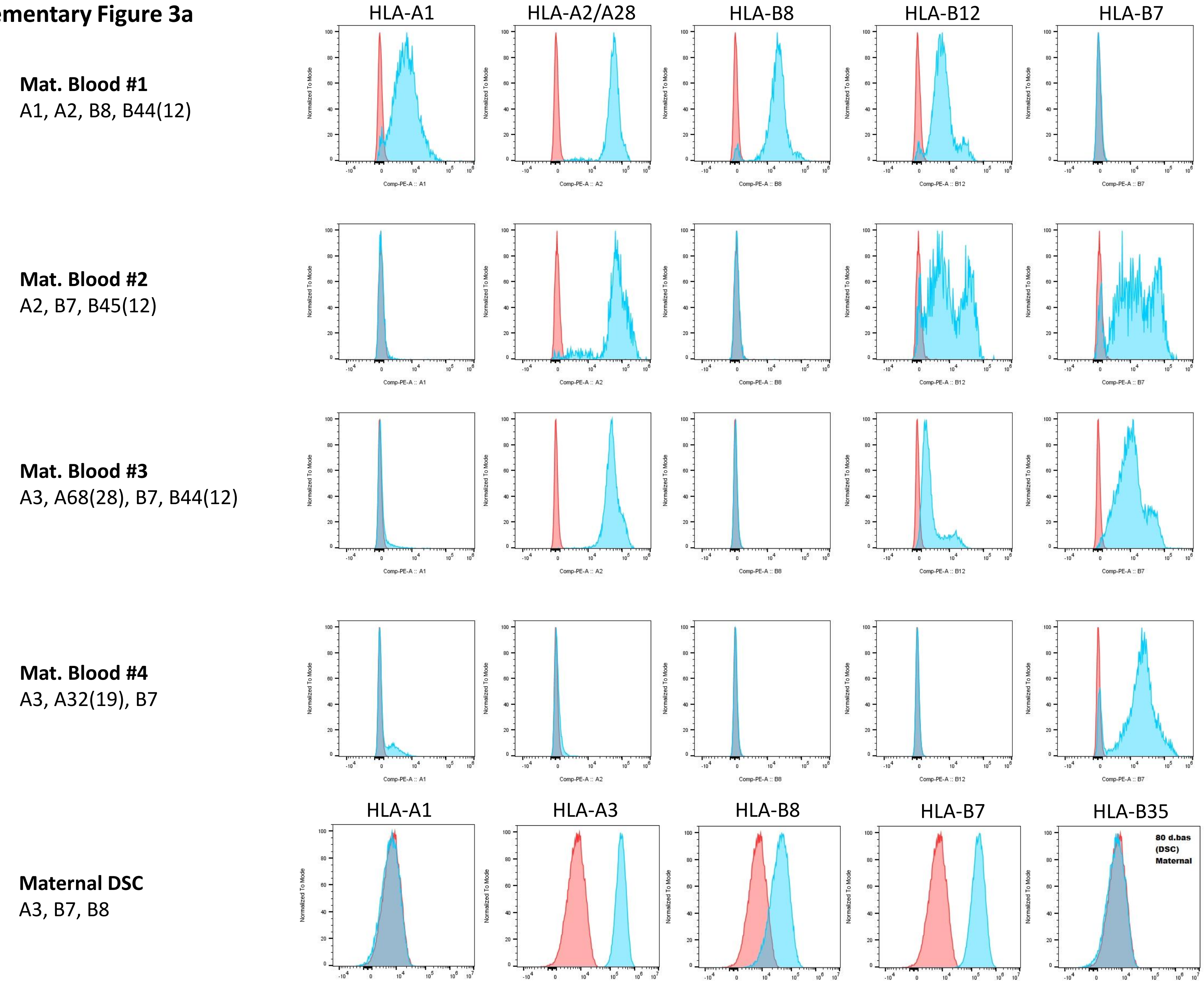

Supplementary Figure 3b

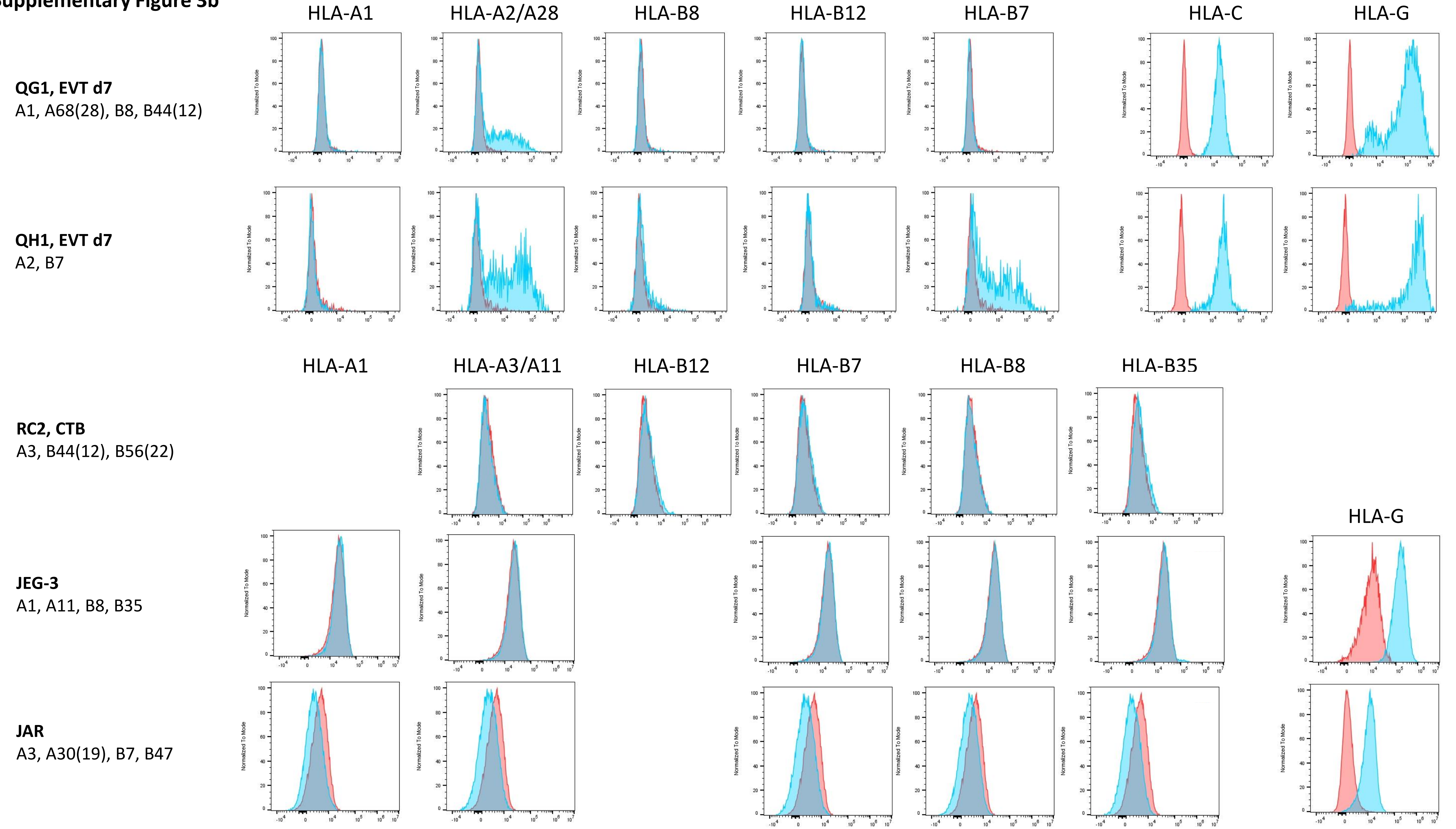

Supplement: Supplementary Figure 1 — Identification of distal cell column trophoblasts in placenta from electively ended pregnancy. (A) Tissue was obtained at first trimester and stained by hematoxylin-eosin (left picture) and for HLA-G (right picture). Proximal- and distal cell column trophoblast have been indicated in the figure. (B) First-trimester placental tissue was stained for HLA-G (left picture) and collagen IV (right picture). [file DataSheet_1.pdf]
