# Supplementary material for: Primary Trophoblast Cultures: Characterization of HLA Profiles and Immune Cell Interactions
Source: Front Immunol. 2022 May 13;13:814019. doi: 10.3389/fimmu.2022.814019 (PMC9136060; doi:10.3389/fimmu.2022.814019)
Supplement: Supplementary Figure 2 — Karyotyping of trophoblast cell lines. Three primary lines (QG1, QH1, RC2) were analyzed at passage 16-17, along with secondary trophoblast lines JAR and JEG-3 as positive controls. No deviations were observed for QH1 (46,XX[5]) and RC2 (46,XY[5]). QG1 was also normal except for a small addition to chromosome 17 (inlay at lower right corner) in two out of five cells (46,XX[3]/46,XX,add(17)(q25)[2]). As comparison, JAR and JEG-3 (not shown) are broadly deviating. [file DataSheet_2.pdf]

Supplementary Table 1

	HLA-A	HLA-B
Mat. Blood #1	02:01	07:02; 45:01
Mat. Blood #2	01:01; 02:01	08:01; 44:02
Mat. Blood #3	03:01; 68:01	07:02; 44:02
Mat. Blood #4	03:01; 32:01	07:02
QG1	01:01; 68:01	08:01; 44:02
QH1	02:01	07:02
RC2	03:01	44:02; 56:01
JEG-3	01:01; 11:01	08:13; 35:01
JAR	03:01; 30:02	07:02; 47:01
